# Altered Auditory Maturation in Fragile X Syndrome and Its Involvement in Audiogenic Seizure Susceptibility

**DOI:** 10.1002/aur.70152

**Published:** 2025-12-15

**Authors:** Dorit Möhrle, Demi Ma, Wenyue Xue, Jun Yan, Ning Cheng

**Affiliations:** ^1^ Hotchkiss Brain Institute, Cumming School of Medicine University of Calgary Calgary Alberta Canada; ^2^ Alberta Children's Hospital Research Institute, Cumming School of Medicine University of Calgary Calgary Alberta Canada; ^3^ Faculty of Veterinary Medicine University of Calgary Calgary Alberta Canada; ^4^ Department of Biological Sciences, Faculty of Science University of Calgary Calgary Alberta Canada; ^5^ Department of Physiology and Pharmacology, Cumming School of Medicine University of Calgary Calgary Alberta Canada

**Keywords:** audiogenic seizures, auditory pathways, *Fmr1* knockout, fragile X syndrome, mice, neurodevelopmental disorders, sensory overload

## Abstract

Auditory hypersensitivity is a prominent symptom in Fragile X syndrome (FXS), the most prevalent monogenic cause of autism and intellectual disability. FXS arises through the loss of the protein encoded by the *FMR1* (Fragile X Messenger Ribonucleoprotein 1) gene, FMRP, required for normal neural circuit excitability. In the brainstem, FMRP is necessary for normal development of acoustic reactivity, and its loss has been implicated in audiogenic seizures (AGS) in *Fmr1* knockout (KO) mice, modeling auditory hypersensitivity and seizures in FXS patients. The present study investigated the correlation between auditory brainstem function and behavioral expression of AGS at the early (postnatal day P20, infancy) and late (P32, juvenile) stages of auditory development in *Fmr1* KO mice compared with wildtype (WT) mice, and in both females and males. We tested responsiveness to pure tones of select auditory pathway elements through auditory brainstem responses, and neural synchronization to amplitude envelopes of modulated acoustic stimuli through auditory steady‐state responses. AGS behavior was categorized for severity during 5‐min exposure to loud sound. Expression of the immediate early gene cFos was quantified as a marker for neuronal activity in the inferior colliculus. During infancy, more severe AGS expression in *Fmr1* KO mice compared with WT mice was accompanied by increased responsiveness to acoustic stimuli at the level of the superior olivary complex and inferior colliculus, and stronger neural synchronicity in subcortical auditory neurons. *Fmr1* KO mice also had higher cFos positive cell counts in the inferior colliculus after exposure to loud sound. With age, both AGS susceptibility and exaggerated acoustic stimulus‐evoked activity in the *Fmr1* KO mice subsided. Intriguingly, *Fmr1* KO mice displayed an altered developmental profile in both the threshold and amplitude of auditory brainstem response. Our findings support evidence that AGS activity relies upon hyperexcitability in the auditory system, including in the lower brainstem, possibly due to disturbed auditory maturation. Hyper‐synchronization to modulated sounds in subcortical auditory neurons seemed to predict AGS severity. The developmental trajectory of the auditory hyperresponsiveness and hypersynchrony suggests a transient processing alteration underlying heightened AGS susceptibility in *Fmr1* KO mice. A better understanding of FXS‐related circuit and behavioral symptoms of auditory processing across development provides the potential to identify therapeutic strategies to achieve auditory function recovery in FXS.

## Lay Summary

Many people with Fragile X syndrome, a genetic condition linked to autism, experience heightened sensitivity to everyday sounds. In this study, we found that young mice with a genetic model of this condition showed stronger responses to sound in certain brain areas involved in hearing, which may explain their heightened reactions to loud noises. These brain responses and sound sensitivity lessened with age, suggesting that early development may be an important period for addressing sound sensitivity through targeted approaches.

**Abbreviations**
A: cerebral aqueduct
ABR: auditory brainstem response
AC: auditory cortex
AGS: audiogenic seizure
AGS1: no response
AGS2: wild running
AGS3: tonic–clonic seizure
AGS4: respiratory arrest
AMPAR: α‐amino‐3‐hydroxy‐5‐methyl‐4‐isoxazole propionic acid receptor
AN: auditory nerve
ART: aligned rank transform
ART‐C: ART contrast tests
ASSR: auditory steady state response
CB: cerebellum
CF: carrier frequency
CN: cochlear nucleus
CUN: cuneiform nucleus
FFT: Fast Fourier transformation
*Fmr1*
: Fragile X messenger ribonucleoprotein 1
FMRP: Fragile X messenger ribonucleoprotein 1
FXS: Fragile X syndrome
HB: Hindbrain
IC: inferior colliculus
ICc: inferior colliculus, central nucleus
ICd: inferior colliculus, dorsal nucleus
ICe: inferior colliculus, external nucleus
KO: knockout
LL: lateral lemniscus
m: medial
MF: modulation frequency
MGB: medial geniculate body
P: postnatal day
PAG: periaqueductal gray
SEM: standard error of the mean
SOC: superior olivary complex
v: ventral
vGlut2: vesicular glutamate transporter 2
WT: wildtype

## 1 Introduction

The brain's ability to process sounds undergoes a tremendous amount of development in early life so that we may hear and communicate effectively. During this period, the auditory system is particularly sensitive to sensory input, with critical windows of enhanced plasticity that vary depending on the complexity of the sounds being processed (Keuroghlian and Knudsen 2007; Kral 2013; Buran et al. 2014; Anbuhl et al. 2022). Critical periods for simple sound features, such as pure frequency tones, occur earlier, whereas more complex sound features undergo distinct periods of plasticity later in development (de Villers‐Sidani et al. 2007; Barkat et al. 2011; Bhumika et al. 2020; Nakamura et al. 2020). This sensitive period‐driven development allows experience to shape the auditory system and its functional capabilities, but disruptions during these periods can have negative, long‐lasting effects on auditory processing (Keuroghlian and Knudsen 2007; Kral 2013). This auditory maturation is commonly altered in neurodevelopmental disorders, such as autism and Fragile X syndrome (FXS, LeBlanc and Fagiolini 2011; Takesian and Hensch 2013; Contractor et al. 2015; Bhumika et al. 2020), which can ultimately lead to auditory processing difficulties (Sinclair et al. 2017).

**FIGURE 1:**
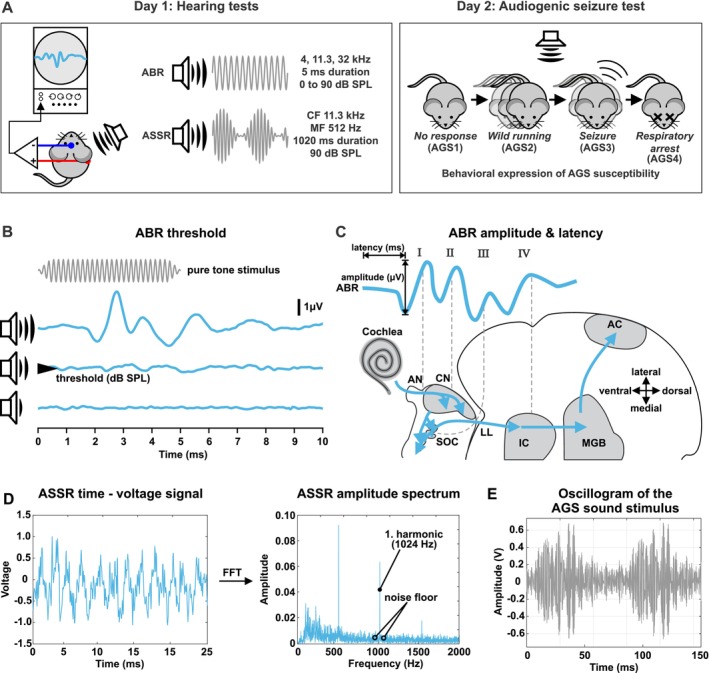
Experimental approach. (A) Timeline including hearing tests (ABR and ASSR measurements) and behavioral expression of AGS susceptibility. (B) Scheme of ABR threshold determination. (C) Schematic drawing of the auditory pathway and correlated stimulus‐evoked deflections of ABR waves I, II, III, and IV. (D) Scheme of ASSR signal‐to‐noise extraction. (E) AGS test stimulus was an amplitude‐ and frequency modulated sound (mean frequency 4.8 kHz, amplitude modulation frequency ~12 Hz). ABR, Auditory Brainstem Response; AC, auditory cortex; AGS, audiogenic seizure; AGS1, no response; AGS2, wild running; AGS3, tonic–clonic seizure; AGS4, respiratory arrest; AN, auditory nerve; ASSR, Auditory Steady State Response; CF, carrier frequency; CN, cochlear nucleus; FFT, Fast Fourier Transformation; IC, inferior colliculus; LL, lateral lemniscus; MF, modulation frequency; MGB, medial geniculate body; SOC, superior olivary complex.

FXS, the leading inherited cause of autism and intellectual disability, is caused by a CGG trinucleotide repeat expansion in the *Fragile X Messenger Ribonucleoprotein 1* (*FMR1*) gene, resulting in the loss of Fragile X Messenger Ribonucleoprotein (FMRP) expression (Fu et al. 1991; Warren and Nelson 1994; Hagerman et al. 2008). A hallmark of both autism and FXS is sensory hypersensitivity and hyperreactivity. Auditory hypersensitivity is one of the most prevalent, persistent, and disabling sensory challenges of FXS and autism, with a majority of affected individuals perceiving everyday sounds as unbearably loud and showing reduced tolerance to sound (Gomes et al. 2008; Sinclair et al. 2017; Rais et al. 2018; Williams, He, et al. 2021; Williams, Suzman, and Woynaroski 2021). These challenges are associated with elevated cortical responses and diminished habituation of the neural response to repeated sounds (Castrén et al. 2003; Knoth et al. 2014; Ethridge et al. 2016; Wang et al. 2017). The *Fmr1* knockout (KO) mouse, a well‐established model for FXS and syndromic autism, recapitulates the human auditory hypersensitivity phenotype in both behavioral and electrical responses (Rotschafer and Razak 2014): *Fmr1* KO mice show hyperreactivity to moderately intense prepulse tones, evidenced by increased prepulse inhibition of the acoustic startle response, and are susceptible to audiogenic seizures (AGS) induced by high‐intensity acoustic stimulation (Musumeci et al. 2000; Chen and Toth 2001; Rotschafer and Razak 2013). AGS in *Fmr1* KO mice involve the ascending auditory pathway and require the inferior colliculus (IC) in the midbrain (Figure 1C; Chen and Toth 2001; Lovelace et al. 2018; Gonzalez et al. 2019; Nguyen et al. 2020). Neuronal hyperresponsiveness in both the auditory cortex and IC, thought to be associated with behavioral hypersensitivity, emerges between the second and third postnatal week corresponding to early auditory development (Wen et al. 2018; Nguyen et al. 2020). Intriguingly, AGS incidence in *Fmr1* KO mice peaks between postnatal days P20 and P30, with particularly heightened susceptibility observed at the beginning of this timeframe (Musumeci et al. 2000; Yan et al. 2005; Dölen et al. 2007; Pacey et al. 2009; Michalon et al. 2012; Nguyen et al. 2020). It has been suggested that lack of FMRP, which is typically highly expressed in the auditory brainstem, leads to dysregulation of critical developmental windows and transient disruptions in auditory brainstem signaling, resulting in age‐dependent hypersensitivity (Yun et al. 2006; Meredith et al. 2012; Wang et al. 2014; Zorio et al. 2017). Yet, despite the clinical significance of auditory hypersensitivity in FXS and other neurodevelopmental disorders, we still lack a clear understanding of the neural mechanisms and temporal maturation dynamics in the auditory brainstem that might contribute to this condition, and this has ultimately hindered efforts to support the affected individuals and families effectively.

In the present study, we investigated the correlation between auditory brainstem function and the behavioral expression of AGS, used as a proxy for auditory hypersensitivity, in *Fmr1* KO mice at two stages of auditory development: early (P18–20, infancy) and late (P31–34, juvenile). We compared behavioral and electrophysiological responses of *Fmr1* KO mice to age‐matched wildtype (WT) controls of both sexes, to assess the effect of altered auditory maturation on brainstem processing and hypersensitivity. Auditory function was assessed through auditory brainstem response (ABR) wave amplitudes and latencies, corresponding to neural responsiveness and transmission speed of distinct parts of the ascending auditory pathway: the auditory nerve (wave I), the cochlear nucleus (wave II), the superior olivary complex (SOC, wave III), and the lateral lemniscus (LL) and IC (wave IV, Figure 1A,C; Melcher and Kiang 1996). Furthermore, we measured auditory steady‐state responses (ASSRs) evoked by amplitude‐modulated sounds to assess the synchronous discharge of subcortical auditory neurons phase‐locked to the amplitude envelope (Figure 1A,D; Kuwada et al. 2002; Picton et al. 2003; Parthasarathy and Bartlett 2012). We found that hyperresponsiveness in the SOC and LL and IC of *Fmr1* KO mice at P20 was associated with a higher propensity for AGS. By P32, both AGS expression and auditory hyperresponsiveness had ceased. A more severe AGS phenotype at P20 in *Fmr1* KO mice also correlated with increased ASSRs to amplitude‐modulated stimuli. These results support evidence that AGS susceptibility is linked to hyperexcitability in the auditory system, including the lower brainstem, possibly due to disrupted maturation during early postnatal development. This temporal pattern highlights that early disruptions in auditory processing during this sensitive period may predispose individuals to auditory hypersensitivity and may indicate a time window when the brain is more susceptible to therapeutic treatments.

## 2 Materials and Methods

### 2.1 Mice

Breeder FVB WT (FVB.129P2‐*Pde6b* ^*+*^ *Tyr* ^*c‐ch*^/AntJ, strain #:004828, RRID: IMSR_JAX:004828) and *Fmr1* KO mice (FVB.129P2‐*Pde6b* ^+^ *Tyr* ^*c‐ch*^ *Fmr1* ^*tm1Cgr*^/J, strain #:004624, RRID: IMSR_JAX:004624) were obtained from the Jackson Laboratory (ME) and the lines were maintained as separate breeding colonies at the mouse facility of the Clara Christie Centre for Mouse Genomics (CCCMG), University of Calgary. WT and *Fmr1* KO animals were not littermates but were age‐matched and maintained on the same background. Mice were group‐housed with up to five mice per cage in a humidity‐ and temperature‐controlled room with a 12‐h light/dark cycle and were fed standard mouse chow *ad libitum*. Hearing measurements were performed at either postnatal day P18–P19 for infants or P31–P33 for juveniles. Behavioral AGS expression was assessed one day after the hearing measurements (Figure 1A). In the following, the age of infants is termed P20 and the age of juveniles P32. Four experimental groups were tested: WT_P20 (*n* = 16; thereof females F = 9, males M = 7), *Fmr1* KO_P20 (*n* = 15; F = 7, M = 8), WT_P32 (*n* = 10; F = 6, M = 4), and *Fmr1* KO_P32 (*n* = 12; F = 6, M = 6). Where indicated, *Fmr1* KO_P20 mice were further stratified for analysis purposes by AGS phenotype: no response (*n* = 4; F = 2, M = 2), wild running (*n* = 3; F = 2, M = 1), tonic–clonic seizure (*n* = 3; F = 2, M = 1), respiratory arrest (*n* = 5; F = 1, M = 4). For immunohistochemical stainings, a separate cohort of animals was used: WT_P20 (*n* = 6; F = 3, M = 3), *Fmr1* KO_P20 (*n* = 7; F = 4, M = 3), WT_P32 (*n* = 6; F = 3, M = 3), and *Fmr1* KO_P32 (*n* = 6; F = 3, M = 3). P20 and P32 mice were from separate cohorts and were not reused across ages. For detailed litter information, see Tables S1 and S2. All procedures in this study were performed in accordance with the recommendations in the Canadian Council for Animal Care. The protocol of this study was approved by the Health Sciences Animal Care Committee of the University of Calgary.

### 2.2 Hearing Measurements

ABRs and ASSRs were recorded in anesthetized mice (Figure 1A, infants: 50 mg/kg ketamine hydrochloride, 2.5 mg/kg xylazine hydrochloride; juveniles: 100 mg/kg ketamine hydrochloride, 5 mg/kg xylazine hydrochloride; intraperitoneal injections) in a soundproof chamber as previously described (Möhrle et al. 2016, 2017; Wolter et al. 2018; Marchetta et al. 2020; Bishop et al. 2022; Abdullah et al. 2025). Anesthesia was supplemented if needed. While differential anesthetic effects cannot be completely ruled out, we observed no obvious indication of greater ABR or ASSR suppression at P20 compared to P32 under ketamine/xylazine. Ketamine‐based anesthesia is commonly used in developmental rodent auditory studies (e.g., Blatchley et al. 1987; Song et al. 2006) and is known to suppress ABR and ASSR potentials compared to awake conditions (van Looij et al. 2004; Sivarao et al. 2016; Nakamura et al. 2023). In vitro studies suggest that ketamine more strongly inhibits NMDA receptor‐mediated currents in immature neurons (e.g., P4–7 vs. P21–28, Jin et al. 2013), raising the possibility of greater suppression in younger animals. However, in vivo evidence on age‐dependent suppression of evoked responses within the age range used in the present study is limited; for example Urch et al. (2001) reported no age‐related differences in nociceptive evoked responses between P14 and P65. Subdermal silver wire electrodes (0.25 mm diameter, annealed; World Precision Instruments AGT1010 or Goodfellow AG00‐WR‐000130) were inserted at the ipsilateral (right) mastoid and the vertex of the animals. Electrodes were custom‐made by soldering silver wire to male 2 mm pin connectors (A‐M Systems) and connected via low‐noise cables (WPI #13620) to the preamplifier (Grass Telefactor P55 AC preamplifier, line filter on). All tests were conducted using a Tucker Davis Technology System 3 (RRID:SCR_014520) with Real‐time processors RP2 and controlled with Jan Schnupp's BrainWare software (V9.21 for TDT). Acoustic stimuli were delivered through a free‐field loudspeaker (MF1, Tucker‐Davis Technologies, Gainesville, FL, USA) positioned 45° lateral to and 20 cm from the right ear of the animal. This angle was chosen to minimize reverberations in our setup and maintain consistency with prior studies in our lab (Bishop et al. 2022; Abdullah et al. 2025). The speaker and animal positions were kept consistent across recordings using table markings and body/pinnae alignment references. Prior to the experiment, the tone amplitude (expressed as dB SPL, reference 20 μPa) of the loudspeaker was calibrated at the same position using a condenser microphone (Model 2520, Larson‐Davis Laboratories, USA) and a microphone preamplifier (Model 2200C, Larson‐Davis Laboratories, USA). Baseline environmental noise was assessed using the TDT saline test protocol, and recordings proceeded only if the noise floor fell within the expected range. Signal quality was verified using BrainWare's Search Mode during sub‐threshold acoustic stimulation, and electrodes were reinserted at the same site as needed (typically ≤ 3 attempts) to minimize noise due to skin contact variability. Conductivity was maintained with regular saline application at the electrode sites. Electrode impedance was not directly measured; however, electrode integrity was ensured through resistance continuity testing, and confirmation of stable, low‐noise signal traces during sub‐threshold stimulation. Between recordings, electrode wires were cleaned with ethanol, gently sanded, and periodically trimmed for reliable skin contact. No online sweep rejection criterion was applied; however, we examined all recordings *post hoc* for evidence of clipping to ensure it did not influence recording outcomes. None of the animals showed evidence of middle ear pathology or obstructive cerumen. Body temperature was maintained at 37°C during testing through a heating pad in the experimental chamber. All recordings were blinded for the subjects' experimental conditions before analysis using custom‐made MATLAB scripts (R2022a, The MathWorks, RRID: SCR_001622).

#### 2.2.1 Auditory Brainstem Response (ABR)

After amplification (50,000‐fold) and filtering (300–3000 Hz), the ABR signals were averaged for 512 repetitions at each sound pressure presented (0–90 dB SPL in steps of 5 dB). ABR thresholds were determined with pure tone stimuli (5 ms, including cosine‐gated 0.5 ms rise/fall time, 4, 11.31, and 32 kHz) as the lowest sound pressure that produces visually distinct evoked potentials (Figure 1B) by an expert observer. These frequencies were chosen based on half‐octave spacing, with 11.3 kHz representing the mid‐frequency and corresponding to the region of best hearing in mice (Song et al. 2006).

ABR waveforms were analyzed for consecutive amplitude deflections (waves, Figure 1C), with each wave consisting of a starting negative peak and the following positive peak. Peak amplitudes and latencies of ABR waves I, II, III, and IV were extracted using custom‐made MATLAB scripts. ABR waveform data were extracted from “.dam” files containing single‐sweep recordings using the “damFileRead” function in MATLAB. For each animal, sweeps (512 per stimulus condition) were averaged for identical stimulus combinations (frequency × dB SPL) over 488 sample points and saved as “.xlsx” files. For each animal and frequency, averaged ABR waveforms were plotted across all sound intensities (e.g., from 90 dB SPL down to 0 dB SPL) to visualize stacked response traces. Peak detection was semi‐automated: initial positive and negative peaks were identified using MATLAB's “findpeaks” function on the averaged waveforms. Candidate peaks were then manually validated using the “ginput” function to select true wave I to IV negative–positive peak pairs starting from the highest SPL. Peak locations (time) and amplitudes were stored using MATLAB's “labeledSignalSet” together with the source data (averaged waveforms) and could be edited using the Signal Labeler app. Extracted peak data were then normalized to individual thresholds (in dB *re* threshold), and peak‐to‐peak amplitudes, latencies, and metadata (e.g., animal ID, genotype, sex, stimulus parameters) were compiled into long‐format “.xlsx” tables for downstream statistical analysis. Wave latencies were defined by the onset timing (leading negative peak) as well as apex (following positive) of each corresponding wave (Figure S1), and ABR wave amplitudes as the peak‐to‐peak difference. From the extracted peaks, ABR peak‐to‐peak (wave) amplitude as well as negative and positive peak latency growth functions were calculated for individual animals for increasing stimulus levels. All ABR wave amplitude and latency growth functions were normalized offline by referencing each animal's individual ABR threshold at each frequency. Specifically, the threshold at a given frequency (e.g., 50 dB SPL at 4 kHz) was set as 0 dB *re* threshold, and all subsequent stimulus levels were expressed relative to that point (e.g., 60 dB SPL became 10 dB *re* threshold). To analyze the slopes of ABR wave amplitude growth functions, we implemented a dynamic thresholding method to identify the response range with amplitude rapid growth (Figure S4). For each individual animal's ABR wave function, we calculated the 99% confidence value across all sound intensities (dB *re* threshold). This value was then used as a horizontal cut‐off to extract data points consistently exceeding this threshold and forming a contiguous growth segment. We then used linear regression to compare the slopes of rapid growth between experimental groups.

#### 2.2.2 Auditory Steady State Response (ASSR)

ASSRs were elicited with amplitude‐modulated sinusoidal stimuli (1020 ms, including cosine‐gated 5 ms rise/fall time) using an 11.31 kHz carrier and modulation frequency of 512 Hz at 90 dB SPL and 100% modulation depth. The signals were filtered (100–10,000 Hz) and averaged for 192 repetitions to compute the ASSR time–domain waveform (Figure 1D). Fast Fourier Transforms were performed on the waveforms starting 20 ms after stimulus onset and ending 25 ms before stimulus offset to exclude transient ABRs and offset responses using custom‐written programs in MATLAB. Specifically, after extracting the waveform data using the “damFileRead” function, sweeps (192 per recording) were averaged over 24,902 sample points and saved as “.xlsx” files. After truncating the time‐domain waveforms to 975 ms, spectral power was determined by Fast Fourier Transformation through MATLAB's “fft” function. No windowing function was applied before Fast Fourier Transformation, consistent with prior rodent ASSR studies (Möhrle et al. 2016; Parthasarathy and Kujawa 2018; Wolter et al. 2018; Marchetta et al. 2022; Savitska et al. 2022). Spectral leakage was minimized by using a long analysis window (975 ms), resulting in ~1 Hz frequency resolution, and the frequency of interest aligned closely with a frequency bin center. We computed the single‐sided spectrum and exported it along with the frequency domains. To calculate the ASSR signal‐to‐noise ratio, we then extracted the maximum amplitude at 1024 Hz (i.e., the first harmonic of the modulation frequency) as well as the amplitude of the noise floor and divided these two values (Figure 1D). The first harmonic (1024 Hz) was chosen over the fundamental frequency (512 Hz) based on studies using the same stimulus parameters (Marchetta et al. 2022; Savitska et al. 2022). In detail, to determine the amplitude of the Fast Fourier Transform peak signal, we examined the frequency bin around 1024 Hz, along with the two adjacent bins above and below it (each 1 Hz wide). The maximum value within these three bins was defined as the signal amplitude. To estimate the noise floor, we excluded the six bins directly adjacent to the signal (three bins above and three bins below), and then averaged the amplitudes from the two three‐bin intervals adjacent to these excluded bins. No cut‐off value was applied to define significance; instead, signal‐to‐noise ratios were treated as continuous variables in line with previous rodent ASSR studies (Möhrle et al. 2016; Wolter et al. 2018; Marchetta et al. 2022; Savitska et al. 2022). The ASSR signal‐to‐noise ratio at the first harmonic of the amplitude modulation frequency was saved along with the metadata for downstream statistical analysis. This recording and analysis approach was adapted from previously published methods (Möhrle et al. 2016; Parthasarathy and Kujawa 2018; Wolter et al. 2018; Marchetta et al. 2022; Savitska et al. 2022).

### 2.3 AGS Testing

To evaluate AGS, mice were placed in a plastic chamber (42 × 42 × 42 cm) which was closed with a sound‐absorbing, fabric‐covered lid containing a personal alarm siren (BOIROS‐type personal security alarm). The siren stimulus was an amplitude‐ and frequency‐modulated sound (mean frequency 4.8 kHz, amplitude modulation frequency ~12 Hz, Figure 1E) and was presented to the mice immediately for a duration of 5 min or until death occurred. The alarm was rewired to operate via a DC power supply to ensure that sound pressure levels of 90 dB SPL were maintained throughout the 5‐min exposure. Mice were categorized for behavioral phenotype based on experimenter observation: AGS1 = no response, AGS2 = wild running, AGS3 = tonic–clonic seizures, AGS4 = respiratory arrest and death as previously described (Dölen et al. 2007; Ronesi et al. 2012). Behavioral scoring of AGS severity was conducted by a single experimenter who was not blinded to genotype. To minimize bias, animals were tested in a consistent order that mirrored the sequence of their hearing assessments the previous day, ensuring comparable recovery times from anesthesia and interleaved experimental groups (genotype and sex). Behavioral observations, including timing and type of response, as well as behaviors unrelated to the sound stimulus (e.g., grooming, exploring, sniffing, rearing), were recorded consistently across all subjects. This approach ensured standardized scoring and yielded results consistent with prior, independent experiments in our laboratory. Mice were given at least 1 h to acclimate to the test room before the behavioral experiment.

### 2.4 Tissue Preparation and Immunohistochemistry

We quantified the expression of the immediate early gene cFos as a marker for neuronal activity in the auditory system (Ehret and Fischer 1991) in a separate cohort of WT_P20 (F = 3, M = 3), *Fmr1* KO_P20 (F = 4, M = 3), WT_P32 (F = 3, M = 3), and *Fmr1* KO_P32 (F = 3, M = 3) mice. The animals were exposed to the AGS test siren for 5 min, and then were deeply anesthetized. The mice were perfused with ice‐cold 0.1 M PBS, pH 7.4 (1X PBS) followed by 4% paraformaldehyde (PFA) in ddH_2_O (~15 min after sound exposure). Brains were extracted, post‐fixed for 48 h in 4% PFA at 4°C, and transferred to 30% sucrose in 1X PBS. Coronal sections (40 μm) containing the IC were cut on a cryostat (Leica CM1850 UV) and kept in 0.02% sodium azide (MKBL4701V, Sigma‐Aldrich) in 1X PBS. After washing three times in Tris‐buffered saline with 0.1% Tween20 detergent (1X TBST; 1, 5, 10 min), the sections were incubated in blocking buffer containing 50 mM glycine in 1X TBS containing 0.1% gelatin, 5% NHS and 0.5% Triton‐X 100 for 90 min at room temperature, and then with primary antibody (c‐Fos (9F6) rabbit mAb #2250, Cell Signaling Technology, 1:1000, RRID:AB_2247211) in antibody solution (0.1% hydrogen peroxide, 0.2% sodium azide, 10 mM glycine in 3% NGS and 0.2% Triton‐X100 in 1X TBS) for 3 days at 4°C. After washing three times with 1X TBST (1, 5, 10 min), the sections were incubated with secondary antibody (Cy3‐conjugated AffiniPure donkey anti‐rabbit IgG (H + L), Jackson ImmunoResearch Laboratories, INC., 1:2000, RRID: AB_2307443) and 4′,6‐diamidino‐2‐phenylindole (DAPI, 1:50) in antibody solution (0.1% hydrogen peroxide, 10 mM glycine in 3% NGS and 0.2% Triton‐X100 in 1X TBS) at room temperature for 2 h. After another three wash cycles in 1X TBST (1, 5, 10 min), sections were mounted with Fluoromount‐G medium (Cat. No.: 0100‐01, SouthernBiotech) on microscope slides (MSSF‐50, FroggaBio) and cover slipped (MCG‐100, FroggaBio). Fluorescent images from the left and right central IC were collected for each animal using an epifluorescence microscope (Olympus BX61) connected to a camera (FLIR Blackfly S BFS‐U3‐16S2M 20523795) and an image capturing software (SpinnakerSDK SpinView 1.10.0.31). We used the open‐source ImageJ/Fiji (RRID:SCR_002285) tool “Quanty‐cFos” (Beretta et al. 2023) to detect cFos positive cells in the microscopic images and derive cell counts (StarDist 2D, Batch Analysis, intensity cut‐off: automatic, area cut‐off: 160, with manual optimization). In more detail, to overcome variability in differentiating cFos positive and negative cells as a result of image brightness, we opted for automated intensity threshold detection in “Quanty‐cFos.” As demonstrated by Beretta et al. (2023) this approach is superior to manually counting cells in that it results in more consistent cFos positive cell counts. To determine the most accurate area cut‐off value, we performed a systematic parameter optimization using a representative subset of 14 images. Each image was independently evaluated by three investigators who manually counted cFos‐positive cells. Their counts were used as a reference to compare the output of three Quanty‐cFos configurations:

1. Intensity cut‐off: automatic, area cut‐off: automatic
2. Intensity cut‐off: automatic, area cut‐off: manually optimized
3. Intensity cut‐off: manually optimized, area cut‐off: manually optimized

Configuration 2 produced results that most closely matched the manual counts. We further refined the area cut‐off in small increments, iteratively testing nearby values until a value of 160 provided the most consistent and accurate detection of cFos‐positive cells with minimal false positives or negatives across the test set of images (≤ 5 cells). After using the tool to detect cFos‐positive cells, all images were reviewed to ensure the correct cell count and the counts were edited if necessary. The cell counts from the left and right IC of each animal were averaged before conducting comparisons between experimental groups.

### 2.5 Statistical Analysis

Statistical tests were performed in GraphPad Prism 9.5.1 (GraphPad Software, San Diego, California, RRID:SCR_002798) and RStudio 2022.2.0.0 (PBC, Boston, Massachusetts, RRID:SCR_000432), and figures were generated in GraphPad Prism and in CorelDRAW X7 (Alludo, Ottawa, Ontario, Canada, RRID:SCR_014235). Prior to statistical testing, datasets were assessed for normality using the D'Agostino and Pearson test or Shapiro–Wilk test for small sample sizes. Datasets that passed normality testing were analyzed using an unpaired *t* test, or ordinary one‐way ANOVA and *post hoc* multiple comparison tests with correction for type 1 error after Dunnett's method. Specifically, parametric tests were used for data presented in Figures 9C and S3. Kruskal‐Wallis and *post hoc* Dunn's multiple comparisons test were used for one‐factor nonparametric datasets (Figure 3B). To accommodate for non‐normal distribution we used ARTool (Aligned Rank Transform, ART) to align‐and‐rank data for nonparametric two‐way, three‐way, and mixed effects ANOVA for main effects and interactions (Wobbrock et al. 2011), and ART‐C for *post hoc* pairwise comparisons (contrast tests, Elkin et al. 2021). All tests, except for data stratified by AGS phenotype and follow‐up ANOVAs, included the effect of sex. *Post hoc* tests (paired for dB *re* threshold) for statistically significant interactions were not significant unless stated otherwise. As a measure for effect sizes, we calculated partial eta squared *η*_p_^2^ for ART ANOVAs, estimates of the difference and standard error for ART‐C tests, the mean difference and 95% confidence interval for *t* tests and Dunnett's tests, and *r* for Dunn's tests. Correlations were tested using Pearson's correlation coefficient for continuous data or Spearman's correlation coefficient for categorized data (*r* < 0.1, negligible; 0.1–0.4, weak; 0.4–0.7, moderate; 0.7–0.9, strong; 0.9–1.0, very strong correlation, Rowntree 2004) and *p* value to quantify the likelihood of a correlation. Simple logistic regression was used for predicting binary outcomes of the AGS test. Statistical significance level was *α* = 0.05, and resulting *p* values are reported in the figure legends and tables using: *p < 0.05; **p < 0.01; ***p < 0.001. Data are presented as group mean and standard error of the mean (SEM) or as group median and interquartile range as stated in the figure legends. Individual data points represent individual animals.

## 3 Results

### 3.1 AGS Susceptibility in *Fmr1* KO Mice Decreases With Age

**FIGURE 2:**
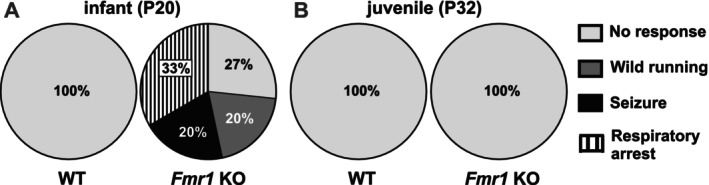
AGS susceptibility in *Fmr1* KO mice decreased with age. (A) During infancy, *Fmr1* KO mice, but not WT mice, displayed a range of behavioral severity in response to loud sound during AGS testing. (B) Both juvenile WT and *Fmr1* KO mice showed no behavioral response to loud sound. Legend applies to (A) and (B): No response (consisting of pause or continuous exploration), wild running, seizure, or respiratory arrest (death). Infants (P20), WT (*n* = 12), *Fmr1* KO (*n* = 16); juveniles (P32), WT (*n* = 10), *Fmr1* KO (*n* = 12). Data expressed as percent of animals.

To determine the effect of auditory maturation on sensory hypersensitivity in FXS, we categorized AGS in both infant (P20) and juvenile (P32) WT and *Fmr1* KO mice. AGS susceptibility was examined by observing the behavioral responses during 5‐min exposure to loud sound (Figure 1A,E). All WT_P20 mice showed no response (100%, Figure 2A) and we observed typical exploratory and self‐grooming behaviors. In comparison, fewer *Fmr1* KO_P20 mice exhibited no response (27%). Consistent with previous findings (Wang et al. 2012; Sawicka et al. 2016; Gonzalez et al. 2019), *Fmr1* KO_P20 mice showed robust AGS phenotypes, with 20% wild running, 20% tonic–clonic seizure, and 33% respiratory arrest (Figure 2A). There was no effect of sex on AGS phenotype in *Fmr1* KO_P20 mice (Table S3). In contrast to infants, both juvenile WT_P32 and *Fmr1* KO_P32 mice had no response (100%, Figure 2B).

These findings indicate that *Fmr1* mutation leads to robust AGS expression during a time that coincides with early auditory development. By the time of late auditory development, AGS susceptibility had subsided.

### 3.2 *Fmr1* Deletion Delays Maturation of Auditory Detection Thresholds

Previous studies suggest a relationship between hearing deficits and AGS severity (Ralls 1967; Faingold et al. 1990). We estimated basic hearing function of infant and juvenile WT and *Fmr1* KO mice using ABR measurements (Figure 1A). We stimulated the auditory system along the low‐to‐high frequency hearing range with pure tone stimuli that allow for the frequency‐specific allocation of auditory responses to a tonotopic place in the cochlea of the auditory periphery (Müller 1991).

**FIGURE 3:**
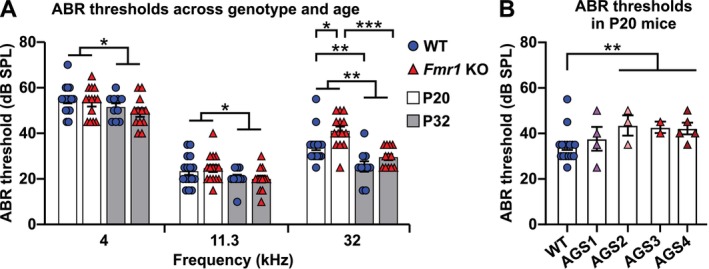
*Fmr1* knock‐out delayed maturation of hearing sensitivity in the high frequency range. (A) Hearing thresholds from infant (P20, white bars) and juvenile (P32, gray bars) WT (blue circles) and *Fmr1* KO mice (red triangles) assessed from electrical ABR potentials in response to low, medium, and high frequency pure tone auditory stimuli (4, 11.3, and 32 kHz). ABR thresholds decreased with age independent of genotype. Additionally, at 32 kHz, ABR thresholds were higher in *Fmr1* KO_P20 compared with WT_P20 mice. (B) ABR thresholds at 32 kHz in WT_P20 (blue circles) and *Fmr1* KO_P20 mice stratified by behavioral phenotype: No response (AGS1, purple triangles), wild running (AGS2, light red), tonic–clonic seizure (AGS3, red), respiratory arrest (AGS4, dark red). Thresholds were significantly higher in *Fmr1* KO mice with AGS2‐4 phenotypes (pooled) compared to WT_P20 mice. Individual AGS2, AGS3, AGS4 groups are shown for completeness, however, no single group differed significantly from WT_P20 when tested independently (Table S6). (A) 4 kHz and 32 kHz, WT_P20 (*n* = 16), *Fmr1* KO_P20 (*n* = 14), WT_P32 (*n* = 10), and *Fmr1* KO_P32 (*n* = 12); 11.3 kHz, WT_P20 (*n* = 16), *Fmr1* KO_P20 (*n* = 15), WT_P32 (*n* = 10), and *Fmr1* KO_P32 (*n* = 12). (B) WT_P20 (*n* = 16), *Fmr1* KO_P20 AGS1 (*n* = 4), AGS2 (*n* = 3), AGS3 (*n* = 2), AGS4 (*n* = 5). Data expressed as mean (bars) ± SEM (error bars) and individual animals (symbols). *p* values, *p < 0.05, **p < 0.01 and ***p < 0.001.

First, we compared ABR thresholds (Figure 1B) to determine if the *Fmr1* mutation led to altered maturation of hearing sensitivity. As expected (Song et al. 2006; Zhou et al. 2006), ABR thresholds varied depending on stimulus frequency (Figure 3A, Table S4). In general, across all experimental animals, thresholds at 11.3 kHz were the lowest (mean ± SEM, 22.5 ± 0.82 dB) and those at 32 kHz (33.4 ± 1.19 dB) were lower than at 4 kHz (52.5 ± 0.90 dB; Figure 3A, Table S5). This means that hearing was most sensitive around the 11.3 kHz region, as is typical for mice (Ralls 1967; Ehret 1974; Heffner and Heffner 2007). The ABR thresholds at all three frequencies decreased with age (Figure 3A, Table 1). A significant influence of genotype was observed only at 32 kHz (Table 1). In particular, ABR thresholds in *Fmr1* KO_P20 mice (41.1 ± 1.9 dB) were higher than in WT_P20 mice (34.4 ± 1.76 dB), whereas those in *Fmr1* KO_P32 mice (29.6 ± 1.3 dB) were similar to WT_P32 mice (25.5 ± 2.29 dB; Figure 3A, Table 2). There was no effect of sex on ABR thresholds (Table S4).

**TABLE 1:** Statistical comparisons of ABR thresholds from mice of the following four groups: WT_P20 (*n* = 16), *Fmr1* KO_P20 (*n* = 14–15), WT_P32 (*n* = 10), and *Fmr1* KO_P32 (*n* = 12).

Parameter	Source of variation	*p*	*p* summary	*F*(DFn, DFd)	*η*_p_^2^
ABR threshold at 4 kHz	Genotype	0.24	ns	*F*(1, 48) = 1.35	0.027
	Age	0.02	*	*F*(1, 48) = 5.43	0.102
	Genotype:Age	0.54	ns	*F*(1, 48) = 0.37	0.008
ABR threshold at 11 kHz	Genotype	0.90	ns	*F*(1, 49) = 0.01	< 0.001
	Age	0.04	*	*F*(1, 49) = 4.10	0.077
	Genotype:Age	0.46	ns	*F*(1, 49) = 0.53	0.011
ABR threshold at 32 kHz	Genotype	0.007	**	*F*(1, 48) = 7.69	0.138
	Age	< 0.001	***	*F*(1, 48) = 28.83	0.375
	Genotype:Age	0.21	ns	*F*(1, 48) = 1.59	0.032

*Note:* The thresholds for one *Fmr1* KO_P20 animal at 4 and 32 kHz were excluded from analysis because the acoustic stimuli were presented in 10 dB instead of 5 dB steps (*n* = 14 for 4 and 32 kHz; *n* = 15 for 11 kHz). This table is associated with data in Figure 3A. Two‐way ART ANOVA, *p* values, *p < 0.05, **p < 0.01, ***p < 0.001; ns, not significant.

**TABLE 2:** *Post hoc* pairwise comparisons for ABR threshold at 32 kHz from mice of the following four groups: WT_P20 (*n* = 16), *Fmr1* KO_P20 (*n* = 14), WT_P32 (*n* = 10), and *Fmr1* KO_P32 (*n* = 12).

Parameter	Contrast	Estimate	SE	DF	*t*	Adjusted *p*	*p* summary
ABR threshold at 32 kHz	WT_P32—WT_P20	−15.66	4.51	48	−3.47	0.005	**
	WT_P32—*Fmr1* KO_P32	−6.63	4.79	48	−1.38	0.51	ns
	WT_P32—*Fmr1* KO_P20	−27.50	4.63	48	−5.94	< 0.001	***
	WT_P20—*Fmr1* KO_P32	9.03	4.27	48	2.11	0.16	ns
	WT_P20—*Fmr1* KO_P20	−11.84	4.10	48	−2.89	0.02	*
	*Fmr1* KO_P32—*Fmr1* KO_P20	−20.88	4.40	48	−4.74	< 0.001	***

*Note:* This table is associated with data in Figure 3A. ART‐C Tukey's multiple comparisons test, *p* values, *p < 0.05, **p < 0.01, ***p < 0.001; ns, not significant.

Hearing sensitivity for high frequencies typically matures later during early postnatal development than for low or middle frequencies (Ehret 1976). In our data, ABR thresholds decreased with age across all frequencies, indicating general maturation of auditory sensitivity. However, a significant effect of the *Fmr1* genotype was observed only at 32 kHz. The higher ABR thresholds at 32 kHz in *Fmr1* KO_P20 mice compared with age‐matched WT controls indicate that the *Fmr1* mutation delayed the maturation of auditory detection thresholds in a frequency‐specific manner.

To further explore a potential association between elevated ABR thresholds and AGS susceptibility, we stratified *Fmr1* KO_P20 mice by behavioral phenotype. *Fmr1* KO_P20 animals were divided into four subgroups as defined in Figure 2: no response (AGS1), wild running (AGS2), tonic–clonic seizure (AGS3), and respiratory arrest (AGS4) and their ABR thresholds were compared to WT_P20 controls. At 32 kHz, a trend towards group differences was observed (Kruskal–Wallis test: *H* = 8.956, *p* = 0.062), whereas no differences were found at 4 kHz (*H* = 0.352, *p* = 0.98) or 11.3 kHz (*H* = 2.733, *p* = 0.60). *Post hoc* comparisons for 32 kHz revealed that the pooled AGS2–4 group had significantly elevated thresholds compared to WT_P20 (Figure 3B, Table 3). This suggests that *Fmr1* KO_P20 mice exhibiting any seizure phenotype, and therefore increased AGS susceptibility, show especially pronounced evidence of delayed auditory maturation relative to WT controls. Notably, there was no correlation between ABR thresholds and AGS severity in *Fmr1* KO_P20 mice at any of the three stimulus frequencies (4 kHz: Pearson r 0.1485, *p* = 0.51; 11.3 kHz: Pearson r 0.2224, *p* = 0.44; 32 kHz: Pearson r 0.1693, *p* = 0.54). This suggests that, while delayed maturation in hearing sensitivity may be more common among AGS‐prone animals, it might not scale directly with AGS severity.

**TABLE 3:** Post hoc pairwise comparisons of ABR thresholds at 32 kHz from mice of the following three groups: WT_P20 (*n* = 16), *Fmr1* KO_P20 AGS1 (*n* = 4), AGS2‐4 (*n* = 10).

Contrast	Mean rank difference	*r*	*Z*	Adjusted *p*	*p* summary
WT P20 vs. AGS2‐4	−10.33	0.59	2.99	0.008	**
WT P20 vs. AGS1	−4.41	0.21	0.92	> 0.99	ns
AGS2‐4 vs. AGS1	5.93	0.31	1.17	0.72	ns

*Note:* This table is associated with data in Figure 3B. Dunn's multiple comparisons test, *p* values, **p < 0.01; ns, not significant.

### 3.3 *Fmr1* Deletion Does Not Affect Maturation of Neuronal Transmission Speed Along the Auditory Pathway

**FIGURE 4:**
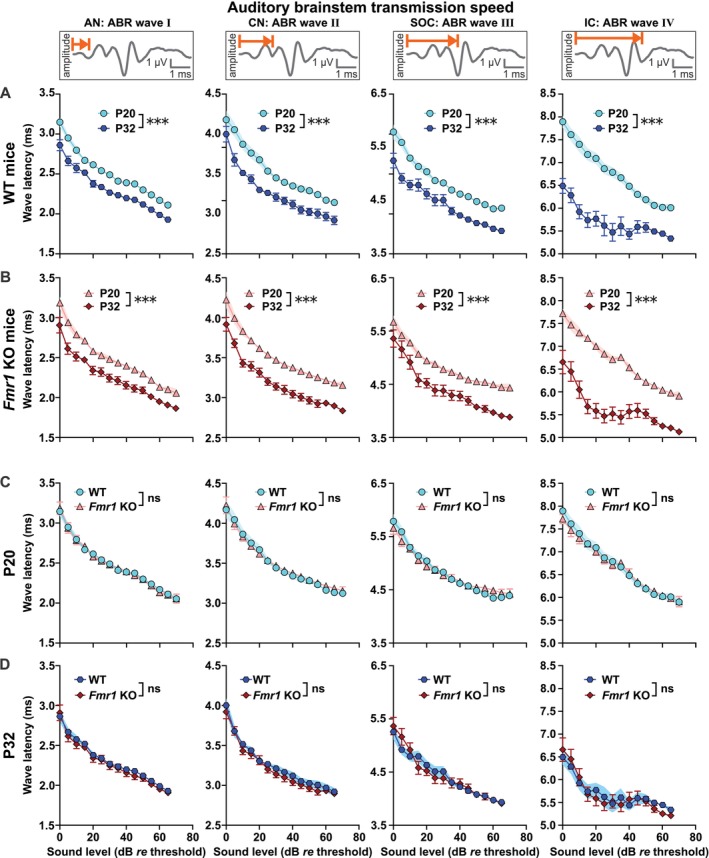
ABR wave I to IV negative peak latencies were similar in WT and *Fmr1* KO mice across development. (A, B) Within‐genotype, between‐age comparisons. (C, D) Within‐age, between‐genotype comparisons. ABR wave latencies corresponding to auditory nerve (AN, wave I), cochlear nucleus (CN, wave II), SOC (wave III), and LL and IC (wave IV) in response to pure tone stimuli (11.3 kHz) with increasing sound intensity. Schematic waveforms for each ABR wave (I–IV) are shown as insets; representative traces are provided in Figure S1. Note that ABR wave latencies are plotted from 0 to 65 or 0 to 70 dB *re* threshold (normalization to ABR threshold). These dB ranges were chosen to include ABRs from at least 3 males and 3 females per experimental group (see Tables S7 and S8 for detailed *n* numbers per group and sound level). ABR wave I–IV latencies decreased with age between (A) WT_P20 (light blue circles) and WT_P32 mice (dark blue hexagons), as well as between (B) *Fmr1* KO_P20 (light red triangles) and *Fmr1* KO_P32 mice (dark red diamonds). ABR wave I to IV latencies were similar between (C) WT_P20 (light blue circles) and *Fmr1* KO_P20 mice (light red triangles), as well as between (D) WT_P32 (dark blue hexagons) and *Fmr1* KO_P32 mice (dark red diamonds). WT_P20 (*n* = 16), *Fmr1* KO_P20 (*n* = 15), WT_P32 (*n* = 10), and *Fmr1* KO_P32 (*n* = 12). Data expressed as mean (symbols) ± SEM (shaded areas or error bars). *p* values, ***p < 0.001, ns not significant.

We analyzed above‐threshold ABR wave level‐latency and ‐amplitude functions corresponding to sound‐evoked neuronal activity in the auditory nerve (wave I), in the cochlear nucleus (wave II), in the SOC (wave III), and in the LL and IC (wave IV; Figure 1C, for exemplary ABR waveforms see Figure S1; Melcher and Kiang 1996). We focused the interpretation of differences in above‐threshold hearing function of *Fmr1* KO_P20, WT_P20, *Fmr1* KO_P32, and WT_P32 mice in the middle frequency range (i.e., 11.3 kHz) because measurement variances were generally smaller for both genotypes. Since elevated ABR thresholds contribute to differences in latencies and amplitudes by reducing the effective stimulus levels, we expressed this analysis as dB *re* ABR threshold (i.e., ABR threshold is 0 dB *re* threshold). ABR wave latencies are known to mature rapidly over the course of the second and third postnatal weeks and to achieve adult‐like characteristics thereafter (Henry 1979; Song et al. 2006). As expected, ABR wave latencies of the leading negative peaks (Figures 4 and S1) were inversely related to sound level, and latencies of later waves were sequentially delayed relative to earlier waves (Figure 4). In WT mice, ABR wave latency growth functions were significantly elevated in WT_P20 compared with WT_P32 mice across the auditory periphery to midbrain (wave I to IV, Figure 4A, Table 4). In line with previous findings (Song et al. 2006), the range over which ABR wave latency values decreased during maturation was larger for the later than for earlier occurring waves. Specifically, on average, wave I latencies decreased by 0.22 ms, wave II by 0.28 ms, wave III by 0.44 ms, and wave IV latencies by 1.08 ms (across 0 to 65 dB *re* threshold). Similar developmental changes were observed in *Fmr1* KO mice (Figure 4B, Table 5): *Fmr1* KO_P20 mice showed significantly longer ABR wave I to IV latencies compared with *Fmr1* KO_P32 mice. Particularly, during maturation, wave I latencies decreased by 0.25 ms, wave II by 0.34 ms, wave III by 0.45 ms, and wave IV latencies by 1.08 ms (across 0 to 70 dB *re* threshold). Interestingly, for ABR wave IV in *Fmr1* KO mice, this developmental decrease in latency was particularly pronounced at near‐threshold to lower sound levels (females: 10 to 40 dB *re* threshold, males: 0 to 35 dB *re* threshold; Table 6).

**TABLE 4:** Statistical comparisons of ABR wave latencies (leading negative peaks, 0 to 65 dB *re* threshold) from WT mice of the following two groups: WT_P20 (*n* = 16) and WT_P32 (*n* = 10).

Parameter	Source of variation	*p*	*p* summary	*F*(DFn, DFd)	*η*_p_^2^
Wave I	Sex	0.9	ns	*F*(1, 21.98) = 0.01	< 0.001
	dB.re.Thr	< 0.001	***	*F*(13, 280.04) = 241.84	0.918
	Age	< 0.001	***	*F*(1, 21.98) = 33.59	0.604
	Sex:dB.re.Thr	0.98	ns	*F*(13, 280.05) = 0.36	0.017
	Sex:Age	0.46	ns	*F*(1, 21.98) = 0.55	0.025
	dB.re.Thr:Age	0.66	ns	*F*(13, 280.05) = 0.79	0.036
	Sex:dB.re.Thr:Age	0.40	ns	*F*(13, 280.05) = 1.05	0.047
Wave II	Sex	0.7	ns	*F*(1, 21.99) = 0.15	0.007
	dB.re.Thr	< 0.001	***	*F*(13, 280.01) = 257.54	0.923
	Age	< 0.001	***	*F*(1, 21.99) = 17.99	0.450
	Sex:dB.re.Thr	0.15	ns	*F*(13, 280.01) = 1.41	0.062
	Sex:Age	0.82	ns	*F*(1, 21.99) = 0.05	0.002
	dB.re.Thr:Age	0.05	ns	*F*(13, 280.01) = 1.72	0.074
	Sex:dB.re.Thr:Age	0.66	ns	*F*(13, 280.01) = 0.8	0.036
Wave III	Sex	0.89	ns	*F*(1, 21.99) = 0.01	< 0.001
	dB.re.Thr	< 0.001	***	*F*(13, 280.03) = 210.28	0.907
	Age	< 0.001	***	*F*(1, 21.99) = 28.55	0.565
	Sex:dB.re.Thr	0.42	ns	*F*(13, 280.03) = 1.03	0.046
	Sex:Age	0.82	ns	*F*(1, 21.99) = 0.05	0.002
	dB.re.Thr:Age	0.11	ns	*F*(13, 280.03) = 1.5	0.065
	Sex:dB.re.Thr:Age	0.40	ns	*F*(13, 280.03) = 1.04	0.046
Wave IV	Sex	0.24	ns	*F*(1, 21.99) = 1.4	0.060
	dB.re.Thr	< 0.001	***	*F*(13, 280.04) = 66.3	0.755
	Age	< 0.001	***	*F*(1, 21.99) = 57.12	0.722
	Sex:dB.re.Thr	0.19	ns	*F*(13, 280.04) = 1.32	0.058
	Sex:Age	0.50	ns	*F*(1, 21.99) = 0.45	0.020
	dB.re.Thr:Age	< 0.001	***	*F*(13, 280.04) = 9.83	0.313
	Sex:dB.re.Thr:Age	0.77	ns	*F*(13, 280.04) = 0.69	0.031

*Note:* Please note that all *post hoc* multiple comparisons for interactions involving sound level (dB *re* threshold) were not significant when matched for sound levels unless stated otherwise. This table is associated with data in Figure 4A. Mixed effects ART ANOVA, *p* values, ***p < 0.001; ns, not significant.

**TABLE 5:** Statistical comparisons of ABR wave latencies (leading negative peaks, 0 to 70 dB *re* threshold) from *Fmr1* KO mice of the following two groups: *Fmr1* KO_P20 (*n* = 15) and *Fmr1* KO_P32 (*n* = 12).

Parameter	Source of variation	*p*	*p* summary	*F*(DFn, DFd)	*η*_p_^2^
Wave I	Sex	0.22	ns	*F*(1, 23.02) = 1.52	0.062
	dB.re.Thr	< 0.001	***	*F*(14, 303.05) = 277.29	0.928
	Age	< 0.001	***	*F*(1, 23.03) = 24.5	0.515
	Sex:dB.re.Thr	0.001	**	*F*(14, 303.06) = 2.63	0.108
	Sex:Age	0.77	ns	*F*(1, 23.02) = 0.08	0.004
	dB.re.Thr:Age	0.09	ns	*F*(14, 303.06) = 1.54	0.067
	Sex:dB.re.Thr:Age	0.83	ns	*F*(14, 303.06) = 0.64	0.029
Wave II	Sex	0.06	ns	*F*(1, 23.02) = 3.69	0.138
	dB.re.Thr	< 0.001	***	*F*(14, 303.04) = 386.07	0.947
	Age	< 0.001	***	*F*(1, 23.03) = 47.99	0.676
	Sex:dB.re.Thr	< 0.001	***	*F*(14, 303.06) = 2.82	0.115
	Sex:Age	0.45	ns	*F*(1, 23.02) = 0.57	0.024
	dB.re.Thr:Age	0.83	ns	*F*(14, 303.06) = 0.63	0.028
	Sex:dB.re.Thr:Age	0.90	ns	*F*(14, 303.06) = 0.54	0.024
Wave III	Sex	0.76	ns	*F*(1, 23.03) = 0.09	0.004
	dB.re.Thr	< 0.001	***	*F*(14, 303.06) = 150.91	0.875
	Age	< 0.001	***	*F*(1, 23.03) = 20.64	0.473
	Sex:dB.re.Thr	< 0.001	***	*F*(14, 303.07) = 3.31	0.133
	Sex:Age	0.33	ns	*F*(1, 23.03) = 0.95	0.040
	dB.re.Thr:Age	0.34	ns	*F*(14, 303.07) = 1.11	0.049
	Sex:dB.re.Thr:Age	0.74	ns	*F*(14, 303.07) = 0.72	0.033
Wave IV	Sex	0.24	ns	*F*(1, 23.06) = 1.45	0.059
	dB.re.Thr	< 0.001	***	*F*(14, 303.18) = 75.18	0.776
	Age	< 0.001	***	*F*(1, 23.05) = 58.3	0.717
	Sex:dB.re.Thr	0.002	**	*F*(14, 303.15) = 2.45	0.102
	Sex:Age	0.86	ns	*F*(1, 23.06) = 0.02	0.001
	dB.re.Thr:Age	< 0.001	***	*F*(14, 303.15) = 9.73	0.310
	Sex:dB.re.Thr:Age	< 0.001	***	*F*(14, 303.16) = 2.71	0.112

*Note:* Please note that all *post hoc* multiple comparisons for interactions involving sound level (dB *re* threshold) were not significant when matched for sound levels unless stated otherwise. This table is associated with data in Figure 4B. Mixed effects ART ANOVA, *p* values, **p < 0.01, ***p < 0.001; ns. not significant.

**TABLE 6:** *Post hoc* pairwise comparisons of ABR wave IV latencies (leading negative peaks, 0–70 dB re threshold) from female or male *Fmr1* KO mice of the following two groups: *Fmr1* KO_P20 (*n* = 15) and *Fmr1 KO*_P32 (*n* = 12).

Contrast	dB	Estimate	SE	DF	*t*	Adjusted *p*	*p* summary
Female *Fmr1* KO_P20 vs. *Fmr1* KO_P32 Sex:dB.re.Thr:Age	0	63.95	30.60	81.55	2.09	0.99	ns
	5	103.12	30.60	81.55	3.37	0.42	ns
	10	146.06	30.60	81.55	4.77	0.009	**
	15	199.63	30.60	81.55	6.52	< 0.001	***
	20	202.33	30.60	81.55	6.61	< 0.001	***
	25	189.63	30.60	81.55	6.20	< 0.001	***
	30	186.64	30.60	81.55	6.10	< 0.001	***
	35	194.11	30.60	81.55	6.34	< 0.001	***
	40	158.74	30.60	81.55	5.19	0.001	**
	45	120.13	30.60	81.55	3.93	0.12	ns
	50	107.87	30.60	81.55	3.52	0.31	ns
	55	126.89	30.60	81.55	4.15	0.06	ns
	60	121.52	30.60	81.55	3.97	0.10	ns
	65	122.17	32.15	96.23	3.80	0.16	ns
	70	135.00	36.26	139.02	3.72	0.18	ns
Male *Fmr1* KO_P20 vs. *Fmr1 KO_P32* Sex:dB.re.Thr:Age	0	173.50	29.71	81.55	5.84	< 0.001	***
	5	140.69	29.71	81.55	4.74	0.01	*
	10	176.83	29.71	81.55	5.95	< 0.001	***
	15	220.17	29.71	81.55	7.41	< 0.001	***
	20	204.75	29.71	81.55	6.89	< 0.001	***
	25	208.79	29.71	81.55	7.03	< 0.001	***
	30	165.81	29.71	81.55	5.58	< 0.001	***
	35	195.40	29.71	81.55	6.58	< 0.001	***
	40	124.88	29.71	81.55	4.20	0.05	ns
	45	107.83	29.71	81.55	3.63	0.25	ns
	50	100.42	29.71	81.55	3.38	0.41	ns
	55	108.91	30.22	86.42	3.60	0.26	ns
	60	114.17	30.22	86.42	3.78	0.17	ns
	65	94.08	31.78	102.09	2.96	0.73	ns
	70	112.45	37.10	159.91	3.03	0.68	ns

*Note:* This table is associated with data in Figure 4B. ART‐C Tukey's multiple comparisons test, *p* values, *p < 0.05, **p < 0.01, ***p < 0.001; ns, not significant.

To assess a possible contribution of altered neuronal transmission speed on AGS susceptibility, we next compared ABR wave latencies of the leading negative peaks between genotypes. In infants, response latencies were similar between WT_P20 and *Fmr1* KO_P20 mice along the ascending auditory pathway (wave I to IV, Figure 4C, Table 7). Similarly, ABR wave latencies were not different in juvenile WT_P32 and *Fmr1* KO_P32 mice (wave I to IV, Figure 4D, Table 8). We further stratified negative peak latency data at 4, 11.3, and 32 kHz of *Fmr1* KO_P20 mice by behavioral AGS phenotype (no response, wild running, seizure, or respiratory arrest). No significant differences were observed between these four AGS subgroups (11.3 kHz: Figure S2; Table S9; 4 and 32 kHz: Tables S10 and S11 for genotype comparisons). This indicates that brainstem conduction is unlikely to underlie differences in seizure phenotype in *Fmr1* KO_P20 mice.

**TABLE 7:** Statistical comparisons of ABR wave latencies (leading negative peaks, 0–70 dB *re* threshold) from infant mice of the following two groups: WT_P20 (*n* = 16) and *Fmr1* KO_P20 (*n* = 15).

Parameter	Source of variation	*p*	*p* summary	*F*(DFn, DFd)	*η*_p_^2^
Wave I	Genotype	0.99	ns	*F*(1, 27.12) = < 0.001	< 0.001
	Sex	0.42	ns	*F*(1, 27.12) = 0.65	0.023
	dB.re.Thr	< 0.001	***	*F*(14, 349.14) = 314.73	0.927
	Genotype:Sex	0.77	ns	*F*(1, 27.12) = 0.08	0.003
	Genotype:dB.re.Thr	0.71	ns	*F*(14, 349.16) = 0.75	0.029
	Sex:dB.re.Thr	0.71	ns	*F*(14, 349.16) = 0.75	0.029
	Genotype:Sex:dB.re.Thr	0.85	ns	*F*(14, 349.16) = 0.61	0.024
Wave II	Genotype	0.55	ns	*F*(1, 27.06) = 0.35	0.013
	Sex	0.76	ns	*F*(1, 27.06) = 0.09	0.003
	dB.re.Thr	< 0.001	***	*F*(14, 349.07) = 377.98	0.938
	Genotype:Sex	0.58	ns	*F*(1, 27.06) = 0.3	0.011
	Genotype:dB.re.Thr	0.58	ns	*F*(14, 349.08) = 0.87	0.034
	Sex:dB.re.Thr	0.58	ns	*F*(14, 349.07) = 0.87	0.034
	Genotype:Sex:dB.re.Thr	0.03	*	*F*(14, 349.07) = 1.78	0.067
Wave III	Genotype	0.84	ns	*F*(1, 27.09) = 0.03	0.001
	Sex	0.58	ns	*F*(1, 27.09) = 0.3	0.011
	dB.re.Thr	< 0.001	***	*F*(14, 349.11) = 248.25	0.909
	Genotype:Sex	0.74	ns	*F*(1, 27.09) = 0.1	0.004
	Genotype:dB.re.Thr	0.02	*	*F*(14, 349.12) = 1.91	0.071
	Sex:dB.re.Thr	< 0.001	***	*F*(14, 349.11) = 2.99	0.107
	Genotype:Sex:dB.re.Thr	0.88	ns	*F*(14, 349.11) = 0.57	0.023
Wave IV	Genotype	0.90	ns	*F*(1, 27.16) = 0.01	< 0.001
	Sex	0.47	ns	*F*(1, 27.16) = 0.53	0.019
	dB.re.Thr	< 0.001	***	*F*(14, 349.19) = 172.66	0.874
	Genotype:Sex	0.81	ns	*F*(1, 27.16) = 0.05	0.002
	Genotype:dB.re.Thr	0.91	ns	*F*(14, 349.2) = 0.53	0.021
	Sex:dB.re.Thr	0.95	ns	*F*(14, 349.21) = 0.46	0.018
	Genotype:Sex:dB.re.Thr	0.12	ns	*F*(14, 349.21) = 1.46	0.055

*Note:* Please note that all *post hoc* multiple comparisons for interactions involving sound level (dB *re* threshold) were not significant when matched for sound levels unless stated otherwise. This table is associated with data in Figure 4C. Mixed effects ART ANOVA, *p* values, *p < 0.05, ***p < 0.001; ns, not significant.

**TABLE 8:** Statistical comparisons of ABR wave latencies (leading negative peaks, 0–65 dB *re* threshold) from juvenile mice of the following two groups: WT_P32 (*n* = 10) and *Fmr1* KO_P32 (*n* = 12).

Parameter	Source of variation	*p*	*p* summary	*F*(DFn, DFd)	*η*_p_^2^
Wave I	Genotype	0.84	ns	*F*(1, 17.99) = 0.03	0.002
	Sex	0.73	ns	*F*(1, 17.99) = 0.11	0.007
	dB.re.Thr	< 0.001	***	*F*(13, 233) = 195.65	0.916
	Genotype:Sex	0.38	ns	*F*(1, 17.99) = 0.8	0.043
	Genotype:dB.re.Thr	0.93	ns	*F*(13, 233) = 0.48	0.026
	Sex:dB.re.Thr	0.007	**	*F*(13, 233) = 2.29	0.114
	Genotype:Sex:dB.re.Thr	0.38	ns	*F*(13, 233) = 1.06	0.056
Wave II	Genotype	0.50	ns	*F*(1, 17.99) = 0.46	0.025
	Sex	0.45	ns	*F*(1, 17.99) = 0.58	0.032
	dB.re.Thr	< 0.001	***	*F*(13, 233) = 231.18	0.928
	Genotype:Sex	0.21	ns	*F*(1, 17.99) = 1.68	0.086
	Genotype:dB.re.Thr	0.73	ns	*F*(13, 233) = 0.73	0.039
	Sex:dB.re.Thr	0.98	ns	*F*(13, 233) = 0.35	0.019
	Genotype:Sex:dB.re.Thr	0.31	ns	*F*(13, 233) = 1.15	0.060
Wave III	Genotype	0.87	ns	*F*(1, 17.99) = 0.02	0.002
	Sex	0.56	ns	*F*(1, 17.99) = 0.35	0.019
	dB.re.Thr	< 0.001	***	*F*(13, 233) = 117.91	0.868
	Genotype:Sex	0.64	ns	*F*(1, 17.99) = 0.22	0.012
	Genotype:dB.re.Thr	0.05	ns	*F*(13, 233) = 1.71	0.088
	Sex:dB.re.Thr	0.57	ns	*F*(13, 233) = 0.87	0.047
	Genotype:Sex:dB.re.Thr	0.06	ns	*F*(13, 233) = 1.68	0.086
Wave IV	Genotype	0.78	ns	*F*(1, 17.99) = 0.07	0.004
	Sex	0.39	ns	*F*(1, 17.99) = 0.75	0.040
	dB.re.Thr	< 0.001	***	*F*(13, 233) = 18.69	0.511
	Genotype:Sex	0.49	ns	*F*(1, 17.99) = 0.49	0.027
	Genotype:dB.re.Thr	0.62	ns	*F*(13, 233) = 0.83	0.044
	Sex:dB.re.Thr	0.009	**	*F*(13, 233) = 2.22	0.110
	Genotype:Sex:dB.re.Thr	0.08	ns	*F*(13, 233.01) = 1.6	0.082

*Note:* Please note that all *post hoc* multiple comparisons for interactions involving sound level (dB *re* threshold) were not significant when matched for sound levels unless stated otherwise. This table is associated with data in Figure 4D. Mixed effects ART ANOVA, *p* values, **p < 0.01, ***p < 0.001; ns, not significant.

**FIGURE 5:**
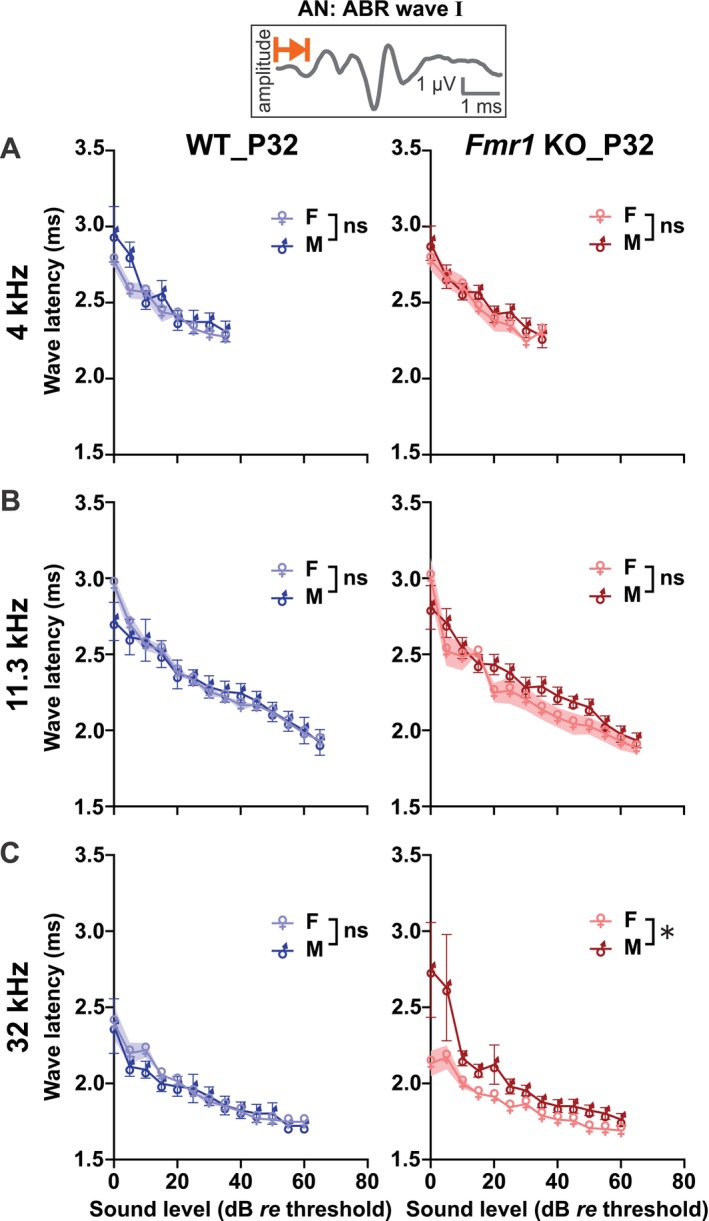
Stimulus frequency‐specific ABR wave I latencies in WT_P32 and *Fmr1* KO_P32 mice. Within‐genotype, between‐sex comparisons of negative peak latencies corresponding to the auditory nerve (AN) in response to pure tone stimuli with increasing sound intensity at (A) 4 kHz (0–35 dB *re* threshold), (B) 11.3 kHz (0–65 dB *re* threshold), (C) 32 kHz (0 to 60 dB *re* threshold). These dB ranges were chosen to include ABRs from at least 3 males and 3 females per experimental group (see Table S8 for detailed *n* numbers per group and sound level). Wave I latencies did not differ between male WT_P32 (dark blue ♂ and line) and female WT_P32 mice (light blue ♀ and line), nor between male *Fmr1* KO_P32 (dark red ♂ and line) and female *Fmr1* KO_P32 mice (light red ♀ and line) at (A) 4 and (B) 11.3 kHz. (C) At 32 kHz, wave I latencies were similar between male and female WT_P32 mice, but significantly longer in male compared to female *Fmr1* KO_P32 mice. WT_P32 (male *n* = 4, female *n* = 6), and *Fmr1* KO_P32 (male *n* = 6, female *n* = 6). Data expressed as mean (symbols) ± SEM (shaded areas or error bars). *p* values, *p < 0.05, ns not significant.

At P32, mice showed a significant genotype × sex interaction for wave I negative latencies at 32 kHz (Table S12). *Post hoc* analyses revealed that *Fmr1* KO_P32 males exhibited significantly longer wave I latencies than *Fmr1* KO_P32 females (Figure 5, Table S13), despite no overall main effect of genotype being present in the full model. This may indicate that the maturation of wave I negative latencies in response to high‐frequency sounds follows a different trajectory in male and female *Fmr1* KO mice. These results underscore the importance of including both sexes in auditory studies, as sex‐specific differences may emerge selectively at certain stimulus frequencies or auditory processing stages.

In line with negative peaks, the following positive peaks were also similar between WT and *Fmr1* KO mice (11.3 kHz: Tables S14 and S15) and between *Fmr1* KO mice sub‐grouped by AGS phenotype (Table S16).

Taken together, our results indicate that *Fmr1* mutation does not substantially alter the maturation of neuronal transmission speed along the auditory brainstem compared to WT controls. Only a subtle, frequency‐dependent effect of sex emerged towards the end of the observed developmental timeframe. Furthermore, our findings suggest that heightened AGS susceptibility during early development is unlikely to be driven exclusively by alterations in neuronal transmission speed.

### 3.4 *Fmr1* Deletion Alters Maturation of Neuronal Responsiveness in Distinct Parts of the Auditory Pathway

Sound‐evoked ABR waveform amplitudes change proportionally to the discharge rate and the number of synchronously firing auditory fibers (Johnson and Kiang 1976). Therefore, changes in above‐threshold ABR waveform amplitudes provide information on the neuronal responsiveness in afferent auditory fibers (Rüttiger et al. 2017). In rodents, the maturation of ABR wave amplitudes occurs non‐monotonically and somewhat independently among the different waves. ABR waves I to III grow during the first ~3–4 postnatal weeks, subsequently decline in amplitude and stabilize at adult values at ~4–5 weeks of age; wave IV shows increased amplitude into the 4th and 5th postnatal week (Henry 1979; Smith and Kraus 1987; Song et al. 2006). To assess whether altered maturation leads to exaggerated auditory neuronal responsiveness in distinct parts of the auditory brainstem and thereby contributes to AGS susceptibility, we analyzed the amplitudes of ABR waves I to IV from *Fmr1* KO_P20, WT_P20, *Fmr1* KO_P32, and WT_P32 mice in response to 11.3 kHz (for exemplary ABR waveforms see Figure S1; *p* values for responses to 4 and 32 kHz are provided in Tables S10–S12).

**FIGURE 6:**
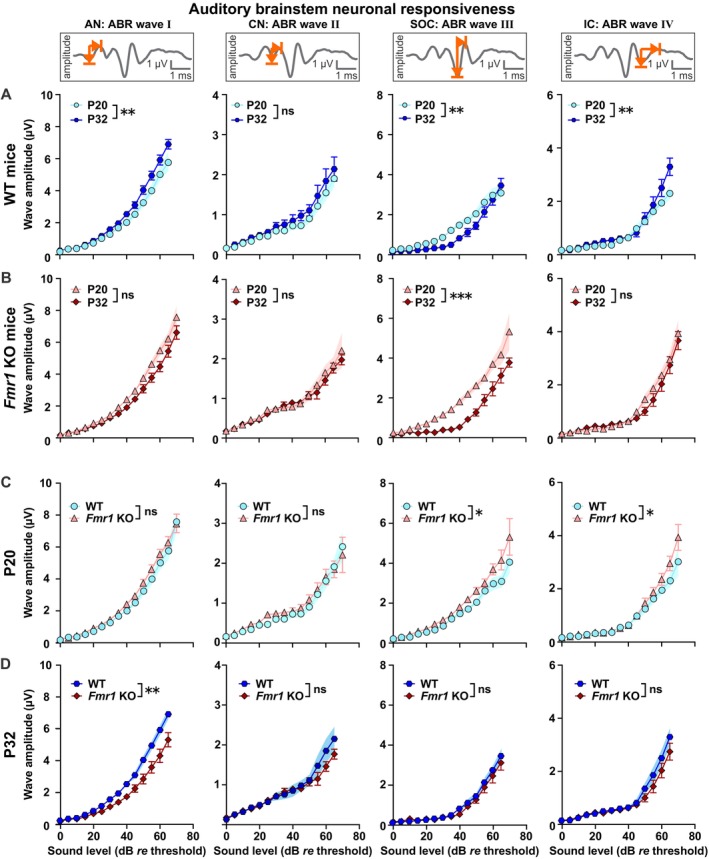
Distinct alterations in ABR wave I to IV peak‐to‐peak amplitudes in *Fmr1* KO mice across development. (A, B) Within‐genotype, between‐age comparisons. (C, D) Within‐age, between‐genotype comparisons. ABR wave amplitudes corresponding to auditory nerve (AN, wave I), cochlear nucleus (CN, wave II), SOC (wave III), and LL and IC (wave IV) in response to pure tone stimuli (11.3 kHz) with increasing sound intensity. Schematic waveforms for each ABR wave (I–IV) are shown as insets; representative traces are provided in Figure S1. Note that ABR wave amplitudes are plotted from 0 to 65 or 0 to 70 dB *re* threshold (normalization to ABR threshold). These dB ranges were chosen to include ABRs from at least 3 males and 3 females per experimental group (see Tables S7 and S8 for detailed *n* numbers per group and sound level). (A) During development in WTs, amplitudes of ABR wave I increased, wave II remained similar, wave III decreased, and wave IV increased in WT_P32 mice (dark blue hexagons) compared to WT_P20 (light blue circles). (B) During development in *Fmr1* KOs, amplitudes of ABR waves I, II, and IV remained similar, and of wave III decreased in *Fmr1* KO_P32 mice (dark red diamonds) compared with *Fmr1* KO_P20 (light red triangles). (C) Between genotypes at P20, amplitudes of ABR wave I and II were similar and of wave III and IV increased in *Fmr1* KO_P20 mice (light red triangles) compared with WT_P20 (light blue circles) mice. (D) Between genotypes at P32, amplitudes of ABR wave I were decreased and of wave II, III, and IV similar in *Fmr1* KO_P32 mice (dark red diamonds) compared with WT_P32 mice (dark blue hexagons). WT_P20 (*n* = 16), *Fmr1* KO_P20 (*n* = 15), WT_P32 (*n* = 10), and *Fmr1* KO_P32 (*n* = 12). Data expressed as mean (symbols) ± SEM (shaded areas or error bars). *p* values, ***p < 0.001, **p < 0.01, *p < 0.05; ns, not significant.

Wave I amplitudes increased with age between WT_P20 and WT_P32 mice (Figure 6A, Table 9), but not between *Fmr1* KO_P20 and *Fmr1* KO_P32 mice (Figure 6B, Table 11). Between‐genotype comparisons demonstrated that wave I amplitudes were similar in infant WT_P20 and *Fmr1* KO_P20 mice (Figure 6C, Table 12), and amplitudes remained significantly lower in juvenile *Fmr1* KO_P32 compared with WT_P32 mice at 11.3 kHz (Figure 6D, Table 13). This indicates that the developmental increase of ABR wave I amplitudes might be delayed or absent in *Fmr1* KO mice. Furthermore, it seems unlikely that sound‐evoked activity in the auditory nerve alone contributes to AGS susceptibility in these mice at the infant age.

**TABLE 9:** Statistical comparisons of ABR wave amplitudes (peak‐to‐peak, 0–65 dB *re* threshold) from WT mice of the following two groups: WT_P20 (*n* = 16) and WT_P32 (*n* = 10).

Parameter	Source of variation	*p*	*p* summary	*F*(DFn, DFd)	*η*_p_^2^
Wave I	Age	0.004	**	*F*(1, 21.99) = 9.92	0.311
	Sex	0.77	ns	*F*(1, 21.99) = 0.08	0.004
	dB.re.Thr	< 0.001	***	*F*(13, 280.02) = 910.94	0.977
	Age:Sex	0.27	ns	*F*(1, 21.99) = 1.23	0.053
	Age:dB.re.Thr	< 0.001	***	*F*(13, 280.03) = 8.72	0.288
	Sex:dB.re.Thr	0.95	ns	*F*(13, 280.02) = 0.43	0.020
	Age:Sex:dB.re.Thr	0.09	ns	*F*(13, 280.02) = 1.55	0.067
Wave II	Age	0.11	ns	*F*(1, 21.99) = 2.63	0.107
	Sex	0.54	ns	*F*(1, 21.99) = 0.36	0.017
	dB.re.Thr	< 0.001	***	*F*(13, 280.03) = 79.83	0.788
	Age:Sex	0.83	ns	*F*(1, 21.99) = 0.04	0.002
	Age:dB.re.Thr	0.02	*	*F*(13, 280.03) = 1.96	0.084
	Sex:dB.re.Thr	0.71	ns	*F*(13, 280.03) = 0.75	0.034
	Age:Sex:dB.re.Thr	0.79	ns	*F*(13, 280.03) = 0.66	0.030
Wave III	Age	0.003	**	*F*(1, 21.98) = 10.95	0.333
	Sex	0.17	ns	*F*(1, 21.99) = 1.98	0.083
	dB.re.Thr	< 0.001	***	*F*(13, 280.03) = 294.54	0.932
	Age:Sex	0.43	ns	*F*(1, 21.99) = 0.62	0.028
	Age:dB.re.Thr	< 0.001	***	*F*(13, 280.04) = 6.25	0.225
	Sex:dB.re.Thr	0.02	*	*F*(13, 280.04) = 1.96	0.084
	Age:Sex:dB.re.Thr	0.39	ns	*F*(13, 280.04) = 1.06	0.047
Wave IV	Age	0.005	**	*F*(1, 21.97) = 9.9	0.311
	Sex	0.63	ns	*F*(1, 21.97) = 0.22	0.010
	dB.re.Thr	< 0.001	***	*F*(13, 280.13) = 85.58	0.799
	Age:Sex	0.49	ns	*F*(1, 21.97) = 0.48	0.021
	Age:dB.re.Thr	0.03	*	*F*(13, 280.12) = 1.88	0.080
	Sex:dB.re.Thr	0.82	ns	*F*(13, 280.12) = 0.63	0.028
	Age:Sex:dB.re.Thr	0.91	ns	*F*(13, 280.12) = 0.52	0.024

*Note:* Please note that all *post hoc* multiple comparisons for interactions involving sound level (dB *re* threshold) were not significant when matched for sound levels unless stated otherwise. This table is associated with data in Figure 6A. Mixed effects ART ANOVA, *p* values, *p < 0.05, **p < 0.01, ***p < 0.001; ns, not significant.

Wave II amplitudes remained similar between WT_P20 and WT_P32 mice (Figure 6A, Table 9), or *Fmr1* KO_P20 and *Fmr1* KO_P32 mice (Figure 6B, Table 11). Amplitudes were also not different in infant WT_P20 and *Fmr1* KO_P20 mice (Figure 6C, Table 12), or juvenile *Fmr1* KO_P32 and WT_P32 mice (Figure 6D, Table 13). Taken together, it appears that the *Fmr1* mutation did not alter wave II amplitude maturation in the observed developmental timeframe. Therefore, summed sound‐evoked activity at the level of the cochlear nucleus likely plays only a minor role in AGS expression.

For ABR wave III, there was a reduction in amplitudes between WT_P20 and WT_P32 mice (Figure 6A, Table 9), as well as between *Fmr1* KO_P20 and *Fmr1* KO_P32 mice (Figure 6B, Table 11). In both WT and *Fmr1* KO mice, this developmental decrease of wave III amplitudes occurred predominantly within sound levels of ~15 to 45 dB *re* threshold (Table 10). On average, this amplitude reduction was more pronounced in *Fmr1* KO (mean ± SEM, 0.79 ± 0.07 μV) compared with WT mice (0.51 ± 0.08 μV, unpaired *t* test, *p* = 0.013, *t* = 2.72, df = 20, mean difference −0.28, 95% confidence interval −0.5021 to −0.06692, Figure S3). Infant *Fmr1* KO_P20 mice showed significantly elevated wave III amplitudes compared to WT_P20 mice (Figure 6C, Table 12). This early genotype difference in ABR responsiveness highlights a potential neural correlate of the heightened AGS susceptibility observed at P20 in *Fmr1* KO mice. By P32, amplitude values in *Fmr1* KO mice had stabilized at WT levels (Figure 6D, Table 13), though there was a difference in sex for ABR wave III amplitudes at P32, with males showing higher amplitudes than females, independent of genotype (Table 13). In summary, these findings indicate that *Fmr1* mutation leads to delayed developmental decrease, that is, maturation, of wave III amplitudes at the infant age. This increased neuronal responsiveness at the level of the SOC during early auditory development might contribute to AGS susceptibility.

**TABLE 10:** *Post hoc* pairwise comparisons of ABR wave III amplitudes (peak‐to‐peak) for contrast age × dB *re* threshold from mice of the following groups: WT_P20 (*n* = 16) and WT_P32 (*n* = 10) or *Fmr1* KO_P20 (*n* = 15) and *Fmr1* KO_P32 (*n* = 12).

Contrast	dB	Estimate	SE	DF	t	Adjusted *p*	*p* summary
WT_P20 vs. WT_P32 Age:dB.re.Thr	0	28.25	17.61	56.02	1.60	0.99	ns
	5	30.55	17.61	56.02	1.73	0.99	ns
	10	40.72	17.61	56.02	2.31	0.84	ns
	15	69.63	17.61	56.02	3.95	0.04	*
	20	67.63	17.61	56.02	3.84	0.06	ns.
	25	81.54	17.61	56.02	4.63	0.005	**
	30	98.31	17.61	56.02	5.58	< 0.001	***
	35	97.69	17.61	56.02	5.55	< 0.001	***
	40	68.95	17.61	56.02	3.91	0.04	*
	45	51.36	17.61	56.02	2.92	0.44	ns
	50	49.90	17.61	56.02	2.83	0.50	ns
	55	36.77	17.61	56.02	2.09	0.93	ns
	60	21.78	17.83	58.61	1.22	0.99	ns
	65	19.78	18.30	64.42	1.08	0.99	ns
*Fmr1* KO_P20 vs. *Fmr1 KO_P32* Age:dB.re.Thr	0	14.22	18.36	78.76	0.77	> 0.99	ns
	5	25.01	18.36	78.76	1.36	0.99	ns
	10	28.57	18.36	78.76	1.56	0.99	ns
	15	84.83	18.36	78.76	4.62	0.004	**
	20	59.59	18.36	78.76	3.25	0.25	ns
	25	112.47	18.36	78.76	6.12	< 0.001	***
	30	98.05	18.36	78.76	5.34	< 0.001	***
	35	113.60	18.36	78.76	6.19	< 0.001	***
	40	103.99	18.36	78.76	5.66	< 0.001	***
	45	81.03	18.36	78.76	4.41	0.009	**
	50	66.55	18.36	78.76	3.62	0.10	ns
	55	42.94	18.51	81.01	2.32	0.87	ns
	60	36.86	18.51	81.01	1.99	0.97	ns
	65	31.36	19.44	95.50	1.61	0.99	ns
	70	31.36	19.44	95.50	1.61	0.99	ns

*Note:* This table is associated with data in Figure 6A,B. ART‐C Tukey's multiple comparisons test, *p* values, *p < 0.05, **p < 0.01, ***p < 0.001; ns, not significant.

ABR wave IV showed increased amplitudes in WT_P32 compared with WT_P20 mice (Figure 6A, Table 9), but no difference between *Fmr1* KO_P32 and *Fmr1* KO_P20 mice (Figure 6B, Table 11). In infancy, *Fmr1* KO_P20 mice had significantly elevated wave IV amplitudes compared with WT_P20 mice (Figure 6C, Table 12). In *Fmr1* KO_P32 mice, amplitude values were similar to WT_P32 levels (Figure 6D, Table 13). This suggests that in *Fmr1* KO mice, the mutation disturbs the timely maturation of wave IV amplitudes, resulting in a premature increase in the response at P20. This exaggerated sound‐evoked activity at the level of the LL and IC might contribute to the heightened AGS expression observed during early auditory development.

**TABLE 11:** Statistical comparisons of ABR wave amplitudes (peak‐to‐peak, 0–70 dB *re* threshold) from *Fmr1* KO mice of the following two groups: *Fmr1* KO_P20 (*n* = 15) and *Fmr1* KO_P32 (*n* = 12).

Parameter	Source of variation	*p*	*p* summary	*F*(DFn, DFd)	*η*_p_^2^
Wave I	Age	0.27	ns	*F*(1, 23.03) = 1.23	0.051
	Sex	0.57	ns	*F*(1, 23.03) = 0.31	0.014
	dB.re.Thr	< 0.001	***	*F*(14, 303.06) = 1158.05	0.982
	Age:Sex	0.18	ns	*F*(1, 23.03) = 1.85	0.075
	Age:dB.re.Thr	< 0.001	***	*F*(14, 303.07) = 4.02	0.157
	Sex:dB.re.Thr	0.83	ns	*F*(14, 303.07) = 0.63	0.028
	Age:Sex:dB.re.Thr	0.27	**	*F*(14, 303.07) = 2.67	0.110
Wave II	Age	0.84	ns	*F*(1, 23.08) = 0.04	0.002
	Sex	0.69	ns	*F*(1, 23.08) = 0.15	0.007
	dB.re.Thr	< 0.001	***	*F*(14, 303.18) = 92.73	0.811
	Age:Sex	0.23	ns	*F*(1, 23.08) = 1.49	0.061
	Age:dB.re.Thr	0.94	ns	*F*(14, 303.2) = 0.46	0.021
	Sex:dB.re.Thr	0.85	ns	*F*(14, 303.2) = 0.61	0.028
	Age:Sex:dB.re.Thr	0.03	*	*F*(14, 303.2) = 1.85	0.079
Wave III	Age	< 0.001	***	*F*(1, 23.07) = 17.98	0.438
	Sex	0.09	ns	*F*(1, 23.06) = 3.09	0.118
	dB.re.Thr	< 0.001	***	*F*(14, 303.12) = 261.59	0.924
	Age:Sex	0.33	ns	*F*(1, 23.06) = 0.96	0.040
	Age:dB.re.Thr	< 0.001	***	*F*(14, 303.15) = 9.65	0.308
	Sex:dB.re.Thr	< 0.001	***	*F*(14, 303.14) = 4.12	0.160
	Age:Sex:dB.re.Thr	0.47	ns	*F*(14, 303.14) = 0.97	0.043
Wave IV	Age	0.60	ns	*F*(1, 23.09) = 0.26	0.012
	Sex	0.34	ns	*F*(1, 23.09) = 0.94	0.039
	dB.re.Thr	< 0.001	***	*F*(14, 303.32) = 95.4	0.815
	Age:Sex	0.88	ns	*F*(1, 23.09) = 0.02	0.001
	Age:dB.re.Thr	0.09	ns	*F*(14, 303.21) = 1.53	0.066
	Sex:dB.re.Thr	0.06	ns	*F*(14, 303.24) = 1.63	0.070
	Age:Sex:dB.re.Thr	0.47	ns	*F*(14, 303.23) = 0.97	0.043

*Note:* Please note that all *post hoc* multiple comparisons for interactions involving sound level (dB *re* threshold) were not significant when matched for sound levels unless stated otherwise. This table is associated with data in Figure 6B. Mixed effects ART ANOVA, *p* values, *p < 0.05, **p < 0.01, ***p < 0.001; ns, not significant.

**TABLE 12:** Statistical comparisons of ABR wave amplitudes (peak‐to‐peak, 0–70 dB *re* threshold) from infant mice of the following two groups: WT_P20 (*n* = 16) and *Fmr1* KO_P20 (*n* = 15).

Parameter	Source of variation	*p*	*p* summary	*F*(DFn, DFd)	*η*_p_^2^
Wave I	Genotype	0.39	ns	*F*(1, 27.1) = 0.74	0.027
	Sex	0.39	ns	*F*(1, 27.09) = 0.75	0.027
	dB.re.Thr	< 0.001	***	*F*(14, 349.09) = 1230	0.980
	Genotype:Sex	0.77	ns	*F*(1, 27.09) = 0.08	0.003
	Genotype:dB.re.Thr	0.04	*	*F*(14, 349.12) = 1.76	0.066
	Sex:dB.re.Thr	0.17	ns	*F*(14, 349.12) = 1.35	0.051
	Genotype:Sex:dB.re.Thr	0.99	ns	*F*(14, 349.12) = 0.27	0.011
Wave II	Genotype	0.45	ns	*F*(1, 27.18) = 0.56	0.020
	Sex	0.38	ns	*F*(1, 27.18) = 0.77	0.028
	dB.re.Thr	< 0.001	***	*F*(14, 349.19) = 98.24	0.798
	Genotype:Sex	0.76	ns	*F*(1, 27.18) = 0.09	0.003
	Genotype:dB.re.Thr	0.47	ns	*F*(14, 349.22) = 0.97	0.038
	Sex:dB.re.Thr	0.008	**	*F*(14, 349.23) = 2.18	0.080
	Genotype:Sex:dB.re.Thr	0.99	ns	*F*(14, 349.23) = 0.29	0.012
Wave III	Genotype	0.02	*	*F*(1, 27.19) = 5.3	0.163
	Sex	0.42	ns	*F*(1, 27.16) = 0.65	0.023
	dB.re.Thr	< 0.001	***	*F*(14, 349.16) = 411.69	0.943
	Genotype:Sex	0.83	ns	*F*(1, 27.16) = 0.04	0.002
	Genotype:dB.re.Thr	< 0.001	***	*F*(14, 349.22) = 3.29	0.117
	Sex:dB.re.Thr	0.12	ns	*F*(14, 349.21) = 1.45	0.055
	Genotype:Sex:dB.re.Thr	0.92	ns	*F*(14, 349.21) = 0.51	0.020
Wave IV	Genotype	0.04	*	*F*(1, 27.3) = 4.43	0.140
	Sex	0.44	ns	*F*(1, 27.29) = 0.59	0.021
	dB.re.Thr	< 0.001	***	*F*(14, 349.51) = 118.99	0.827
	Genotype:Sex	0.64	ns	*F*(1, 27.29) = 0.21	0.008
	Genotype:dB.re.Thr	0.002	**	*F*(14, 349.4) = 2.49	0.091
	Sex:dB.re.Thr	0.06	ns	*F*(14, 349.39) = 1.64	0.062
	Genotype:Sex:dB.re.Thr	0.91	ns	*F*(14, 349.38) = 0.52	0.021

*Note:* Please note that all *post hoc* multiple comparisons for interactions involving sound level (dB *re* threshold) were not significant when matched for sound levels unless stated otherwise. This table is associated with data in Figure 6C. Mixed effects ART ANOVA, *p* values, *p < 0.05, **p < 0.01, ***p < 0.001; ns, not significant.

**TABLE 13:** Statistical comparisons of ABR wave amplitudes (peak‐to‐peak, 0–65 dB *re* threshold) from juvenile mice of the following two groups: WT_P32 (*n* = 10) and *Fmr1* KO_P32 (*n* = 12).

Parameter	Source of variation	*p*	*p* summary	*F*(DFn, DFd)	*η*_p_^2^
Wave I	Genotype	0.005	**	*F*(1, 21.97) = 9.9	0.462
	Sex	0.63	ns	*F*(1, 21.97) = 0.22	0.112
	dB.re.Thr	< 0.001	***	*F*(13, 280.13) = 85.58	0.980
	Genotype:Sex	0.49	ns	*F*(1, 21.97) = 0.48	0.028
	Genotype:dB.re.Thr	0.03	*	*F*(13, 280.12) = 1.88	0.481
	Sex:dB.re.Thr	0.82	ns	*F*(13, 280.12) = 0.63	0.095
	Genotype:Sex:dB.re.Thr	0.91	ns	*F*(13, 280.12) = 0.52	0.083
Wave II	Genotype	0.33	ns	*F*(1, 17.99) = 1.001	0.053
	Sex	0.53	ns	*F*(1, 17.99) = 0.39	0.021
	dB.re.Thr	< 0.001	***	*F*(13, 233) = 73.32	0.804
	Genotype:Sex	0.33	ns	*F*(1, 17.99) = 0.99	0.052
	Genotype:dB.re.Thr	0.04	*	*F*(13, 233) = 1.82	0.092
	Sex:dB.re.Thr	0.85	ns	*F*(13, 233) = 0.59	0.032
	Genotype:Sex:dB.re.Thr	0.55	ns	*F*(13, 233) = 0.89	0.048
Wave III	Genotype	0.82	ns	*F*(1, 17.99) = 0.05	0.003
	Sex	0.009	**	*F*(1, 17.99) = 8.45	0.320
	dB.re.Thr	< 0.001	***	*F*(13, 233) = 115.71	0.866
	Genotype:Sex	0.49	ns	*F*(1, 17.99) = 0.47	0.026
	Genotype:dB.re.Thr	0.98	ns	*F*(13, 233) = 0.32	0.018
	Sex:dB.re.Thr	< 0.001	***	*F*(13, 233) = 5.35	0.230
	Genotype:Sex:dB.re.Thr	0.23	ns	*F*(13, 233) = 1.25	0.066
Wave IV	Genotype	0.31	ns	*F*(1, 17.99) = 1.06	0.056
	Sex	0.77	ns	*F*(1, 17.99) = 0.08	0.005
	dB.re.Thr	< 0.001	***	*F*(13, 233.02) = 59.89	0.770
	Genotype:Sex	0.21	ns	*F*(1, 17.99) = 1.62	0.083
	Genotype:dB.re.Thr	0.73	ns	*F*(13, 233.02) = 0.72	0.039
	Sex:dB.re.Thr	0.35	ns	*F*(13, 233.02) = 1.1	0.058
	Genotype:Sex:dB.re.Thr	0.55	ns	*F*(13, 233.02) = 0.9	0.048

*Note:* Please note that all *post hoc* multiple comparisons for interactions involving sound level (dB *re* threshold) were not significant when matched for sound levels unless stated otherwise. This table is associated with data in Figure 6D. Mixed effects ART ANOVA, *p* values, *p < 0.05, **p < 0.01, ***p < 0.001; ns, not significant.

**FIGURE 7:**
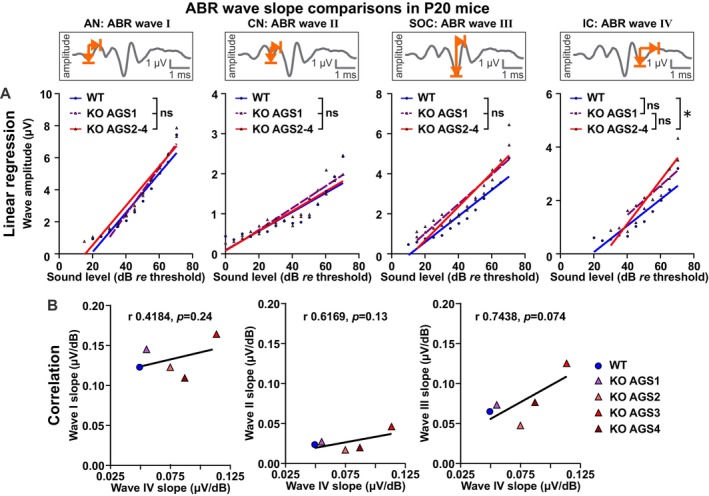
Slope analysis of ABR wave amplitude growth functions in P20 mice depending on AGS susceptibility. (A) The dynamic ranges of ABR wave I, II, III, and IV level‐amplitude functions (dB range of rapid amplitude growth) were extracted for individual animals based on the 99% confidence values and pooled for mice depending on AGS phenotype. Slopes of ABR wave growth function were compared between WT_P20 controls (blue line), *Fmr1* KO_P20 AGS1 (no response; purple dashed line), and *Fmr1* KO_P20 AGS2‐4 mice (wild running, seizure, respiratory arrest; red line) using linear regression. Slopes were not significantly different between the three groups for ABR wave I (Table 14, WT_P20: *Y* = 0.1229 × *X* − 2.324, *R* ^2^ 0.9288; *Fmr1* KO_P20 AGS1: *Y* = 0.1452 × *X* − 3.317, *R* ^2^ 0.9734; *Fmr1* KO_P20 AGS2‐4: *Y* = 0.1231 × *X* − 1.901, *R* ^2^ 0.9386), wave II (Table 14, WT_P20: *Y* = 0.02377 × *X* + 0.09261, *R* ^2^ 0.7800; *Fmr1* KO_P20 AGS1: *Y* = 0.02728 × *X* + 0.05241, *R* ^2^ 0.8644; *Fmr1* KO_P20 AGS2‐4: *Y* = 0.02464 × *X* + 0.08063, *R* ^2^ 0.8488), and wave III (Table 14, WT_P20: *Y* = 0.06513 × *X* − 0.7204, *R* ^2^ 0.9082; *Fmr1* KO_P20 AGS1: *Y* = 0.07350 × *X* − 0.4503, *R* ^2^ 0.8862; *Fmr1* KO_P20 AGS2‐4: *Y* = 0.08416 × *X* − 1.008, *R* ^2^ 0.8634). Slopes of ABR wave IV amplitude growth functions were significantly different (Table 14, WT_P20: *Y* = 0.04939 × *X* − 0.9198, *R* ^2^ 0.8524; *Fmr1* KO_P20 AGS1 *Y* = 0.05520 × *X* − 0.7538, *R* ^2^ 0.8429; *Fmr1* KO_P20 AGS2‐4: *Y* = 0.08171 × *X* − 2.139, *R* ^2^ 0.8797), in particular between WT_P20 and *Fmr1* KO_P20 AGS2‐4 mice (Table 14). Slopes between WT_P20 and *Fmr1* KO_P20 AGS1 as well as *Fmr1* KO_P20 AGS1 and *Fmr1* KO_P20 AGS2‐4 mice were not different (Table 14). (B) Slopes of ABR wave IV correlated with slopes of wave I (left), II (middle), or III (right) amplitude growth functions from WT_P20 (blue circles), *Fmr1* KO_P20 AGS1 (no response, purple triangles), AGS2 (wild running, light red triangles), AGS3 (seizure, red triangles), and AGS4 (respiratory arrest, dark red triangles) mice. Slopes of wave I and wave II showed no statistically significant correlation with wave IV (wave I v IV: Pearson r 0.4184, one‐tailed *p* = 0.24; II v IV: Pearson r 0.6169, one‐tailed *p* = 0.13). Slopes of wave III and IV showed a strong positive correlation that reached a statistical trend (wave III v IV: Pearson r 0.7438, one‐tailed *p* = 0.074). (A) WT_P20 (*n* = 16), *Fmr1* KO_P20 AGS1 (*n* = 4), KO_P20 AGS2‐4 (*n* = 11), (B) WT_P20 (*n* = 16), *Fmr1* KO_P20 AGS1 (*n* = 4), KO_P20 AGS2 (*n* = 3), KO_P20 AGS3 (*n* = 3), KO_P20 AGS4 (*n* = 5). Data expressed as mean (symbols) and linear regression lines. Regression lines in (B) are only for visual clarity. *p* values, *p < 0.05; ns, not significant.

To further assess the contribution of increased neuronal responsiveness across the ascending auditory pathway to AGS, we next pooled the ABR wave amplitudes of infants into three groups based on AGS phenotypes, that is, WT_P20 (all with no response), *Fmr1* KO_P20 with no response (AGS1), or *Fmr1* KO_P20 mice with any response (AGS2‐4, including wild running, seizure, and respiratory arrest). We extracted the dynamic range of wave level‐amplitude functions (dB range of rapid amplitude growth) for individual animals based on the 99% confidence values (Figure S4). We then compared the slopes of ABR wave I to IV amplitude growth functions using linear regression as a measure for the efficiency and sensitivity of the neural response to increasing sound intensity. The slopes of either ABR wave I and II growth functions were not significantly different between the three groups (Figure 7A, Table 14, wave I, WT_P20: *Y* = 0.1229 × *X* − 2.324, *Fmr1* KO_P20 AGS1: *Y* = 0.1452 × *X* − 3.317, *Fmr1* KO_P20 AGS2‐4: *Y* = 0.1231 × *X* − 1.901; wave II, WT_P20: *Y* = 0.02377 × *X* + 0.09261, *Fmr1* KO_P20 AGS1: *Y* = 0.02728 × *X* + 0.05241, *Fmr1* KO_P20 AGS2‐4: *Y* = 0.02464 × *X* + 0.08063). Surprisingly, the slopes of ABR wave III were also not significantly different, though there was a slight increase in steepness with AGS susceptibility (Figure 7A, Table 14, WT_P20: *Y* = 0.06513 × *X* − 0.7204, *Fmr1* KO_P20 AGS1: *Y* = 0.07350 × *X* − 0.4503, *Fmr1* KO_P20 AGS2‐4: *Y* = 0.08416 × *X* − 1.008). In contrast to waves I to III, the slopes of wave IV amplitude growth functions in *Fmr1* KO_P20 mice with any AGS response were significantly steeper than in WT_P20 mice (Figure 7A, Table 14, WT_P20: *Y* = 0.04939 × *X* − 0.9198, *Fmr1* KO_P20 AGS2‐4: *Y* = 0.08171 × *X* − 2.139), whereas those in *Fmr1* KO_P20 mice with no response in the AGS test were not different (Table 14, *Y* = 0.05520 × *X* − 0.7538). This indicates that the auditory midbrain at the level of the LL and IC in *Fmr1* KO mice with AGS responded more intensely or showed a more rapid increase in response amplitude to changes in sound level compared to WT mice, which did not exhibit any AGS response.

**TABLE 14:** Statistical comparisons of ABR wave amplitude growth slopes (dB range of rapid amplitude increase) in P20 mice grouped by AGS phenotype: WT_P20 (*n* = 16), *Fmr1* KO_P20 AGS1 (no response; *n* = 4), and *Fmr1* KO_P20 AGS2‐4 (wild running, seizure, respiratory arrest; *n* = 11).

Parameter	Comparison	*p*	*p* summary	*F*(DFn, DFd)
Wave I	WT_P20 vs. AGS1 vs. AGS2‐4	0.37	ns	*F*(2, 26) = 1.023
Wave II	WT_P20 vs. AGS1 vs. AGS2‐4	0.76	ns	*F*(2, 37) = 0.2705
Wave III	WT_P20 vs. AGS1 vs. AGS2‐4	0.27	ns	*F*(2, 31) = 1.331
Wave IV	WT_P20 vs. AGS1 vs. AGS2‐4	0.04	*	*F*(2, 21) = 3.702
	WT_P20 vs. AGS1	0.68	ns	*F*(1, 14) = 0.1690
	WT_P20 vs. AGS2‐4	0.022	*	*F*(1, 16) = 6.408
	AGS1 vs. AGS2‐4	0.15	ns	*F*(1, 12) = 2.278

*Note:* This table is associated with data in Figure 7A. Linear regression, *p* values, *p < 0.05; ns, not significant.

We next aimed to determine if this hyperresponsiveness originated in the midbrain, or if it may be relayed from the lower auditory structures. To this end, we correlated the slopes of ABR wave I, II, or III amplitude growth functions with those of wave IV from WT_P20, *Fmr1* KO_P20 AGS1, 2, 3, and 4 mice (Figure 7B). We assumed that if there was no or weak correlation, then the hyperresponsiveness originated in the midbrain. If there was a strong positive correlation, then the hyperresponsiveness may be relayed from lower auditory structures. Neither the slope of wave I nor the slope of wave II showed a statistically significant correlation with the slope of wave IV (Figure 7B, wave I v IV: Pearson r 0.4184, one‐tailed *p* = 0.24; II v IV: Pearson r 0.6169, one‐tailed *p* = 0.13). The strong correlation between the slopes of waves III and IV reached a statistical trend (Figure 7B, wave III v IV: Pearson r 0.7438, one‐tailed *p* = 0.074). These results suggest that the auditory hyperresponsiveness contributing to heightened AGS vulnerability in *Fmr1* KO mice may originate, at least in part, in the lower auditory brainstem and then be relayed to and pathologically amplified in the auditory midbrain.

Taken together, these results indicate that *Fmr1* plays an important role in the proper maturation of neuronal responsiveness in distinct parts of the ascending auditory pathway, particularly at the level of the auditory nerve, SOC, as well as the LL and IC. Among these, increased responsiveness in the SOC as well as LL and IC during early auditory development may underlie transient AGS susceptibility in *Fmr1* KO mice and contribute to sensory hypersensitivity in FXS.

### 3.5 Increased Number of cFos Positive Cells in the IC of in *Fmr1* KO Mice

**FIGURE 8:**
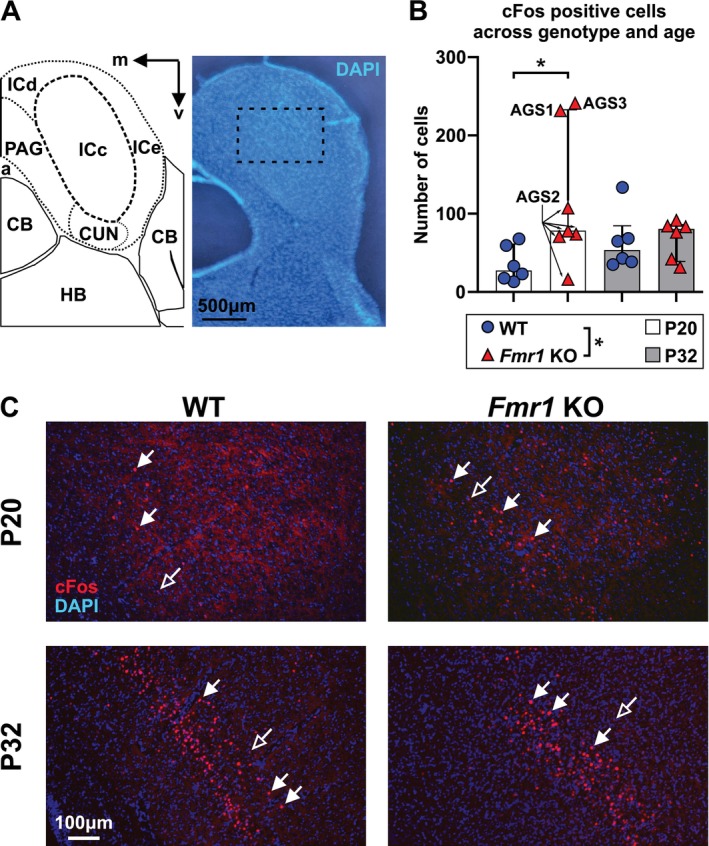
Number of cFos positive cells in the IC was increased in infant *Fmr1* KO mice. (A) Schematic overview and coronal section of a mouse brain showing the IC. Cell nuclei were stained with DAPI (blue), and the dashed rectangle indicates the region where the IC was imaged at higher magnification for cFos quantification. Please note that the cerebellum was incomplete in the coronal section. (B) Quantification of cFos immunopositive cells in the IC of infant (white bars) or juvenile (gray bars) WT (blue circles) and *Fmr1* KO (red triangles) mice. The number of cFos positive cells was significantly different between genotypes. In particular, the cFos positive cell density was higher in *Fmr1* KO_P20 compared with WT_P20 mice. (C) Representative coronal brain sections containing the IC stained with antibodies against cFos (red). Immunopositive dots were counted to estimate the number of cFos positive cells in the central nucleus of the IC. The effect of *Fmr1* mutation on the number of cFos positive cell counts were analyzed in infant (P20, upper panel) and juvenile (P32, lower panel) WT (left) and *Fmr1* KO mice (right) after exposure to the same sound used in the AGS test. Filled arrows indicate cFos positive cells and open arrows indicate cFos negative cells. Cell nuclei were stained with DAPI (blue). Scale bars, (A) 500 μm, (C) 100 μm. AGS1 = no response, AGS2 = wild running, AGS 3 = seizure. WT_P20 (*n* = 6), *Fmr1* KO_P20 (*n* = 7), WT_P32 (*n* = 6), and *Fmr1* KO_P32 (*n* = 6). Data expressed as median (bars) ± interquartile range (error bars) and individual animals (symbols). *p* value, *p < 0.05. A, cerebral aqueduct; CB, cerebellum; CUN, cuneiform nucleus; HB, hindbrain; ICc, inferior colliculus, central nucleus; ICd, inferior colliculus, dorsal nucleus; ICe, inferior colliculus, external nucleus; m, medial; PAG, periaqueductal gray; v, ventral.

cFos expression has been widely used as a reliable histological marker to map neuronal activity in the central nervous system, including stimulus‐related responses on a single‐cell level in the auditory pathway (Ehret and Fischer 1991; Yang et al. 2005; Moreno‐Paublete et al. 2017). Previous studies have suggested that an increase in sound‐evoked cFos‐positive cell density in specific auditory nuclei might be a correlate of sensory hypersensitivity in early development (Chen and Toth 2001; Nguyen et al. 2020; Yang et al. 2020). We stained coronal brain sections containing the IC from a separate cohort of infant and juvenile WT and *Fmr1* KO mice to determine if hyperresponsiveness in the auditory midbrain during early development is associated with increased cFos expression. Before brain extraction, the animals from both genotypes and age groups were sound‐exposed to the same siren as in the AGS test (Figure 1E). Microscopic images of the central nucleus of the IC (Figure 8A), in an area assumed to represent a ~8–40 kHz sound frequency region (Ryugo and Milinkeviciute 2023), were collected and analyzed for cFos immunoreactive labelling (Figure 8C) in a semi‐automated fashion using the ImageJ/Fiji tool “Quanty‐cFos” (Beretta et al. 2023). cFos‐positive cell counts from WT_P20, *Fmr1* KO_P20, WT_P32, and *Fmr1* KO_P32 mice showed a genotype effect (Figure 8B, Table 15), with a higher cFos‐positive cell density in *Fmr1* KO mice (median ± interquartile range, 77.5 ± 56.0–99.2) compared with WT mice (40.3 ± 25.0–66.8). *Post hoc* comparisons showed that this difference was particularly pronounced between *Fmr1* KO_P20 (77.5 ± 70.5–232.0) and WT_P20 mice (27.5 ± 15.9–61.1; Figure 8B, Table 16). In contrast to infant mice, the difference was not statistically significant in juvenile *Fmr1* KO_P32 (79.5 ± 38.9–85.8) and WT_P32 mice (53.5, ±37.1–84.4). Though the interaction between age × genotype of cFos‐positive cell counts was not significant (Figure 8B, Table 15), it is worth noting that the direction of age‐related changes in cFos labelling matched those of ABR wave IV amplitudes in either WT and *Fmr1* KO mice: between P20 and P32 both cFos‐positive cell density and ABR wave IV amplitudes increased in WT mice (Figures 6A and 8B), whereas both remained similar in *Fmr1* KO mice (Figures 6B and 8B). Interestingly, we did not observe a systematic pattern in cFos expression in *Fmr1* KO_P20 mice, regardless of whether they showed no behavioral response in the AGS test or exhibited any response (here including wild running and seizure, Figure 8B). However, this observation should be interpreted with caution due to the small sample size. There was no effect of sex on cFos‐positive cell counts (Table 15).

**TABLE 15:** Statistical comparisons of number of cFos positive cells from mice of the following four groups: WT_P20 (*n* = 6), *Fmr1* KO_P20 (*n* = 7), WT_P32 (*n* = 6), and *Fmr1* KO_P32 (*n* = 6).

Parameter	Source of variation	*p*	*p* summary	*F*(DFn, DFd)	*η*_p_^2^
cFos positive cells	Sex	0.77	ns	*F*(1, 17) = 0.08	0.005
	Genotype	0.04	*	*F*(1, 17) = 4.45	0.208
	Age	0.94	ns	*F*(1, 17) = 0.004	> 0.001
	Sex:Genotype	0.75	ns	*F*(1, 17) = 0.1	0.006
	Sex:Age	0.86	ns	*F*(1, 17) = 0.02	0.002
	Genotype:Age	0.11	ns	*F*(1, 17) = 2.78	0.141
	Sex:Genotype:Age	0.59	ns	*F*(1, 17) = 0.29	0.017

*Note:* This table is associated with data in Figure 8B. Three‐way ART ANOVA, *p* values, *p < 0.05; ns, not significant.

**TABLE 16:** *Post hoc* pairwise comparisons of number of cFos positive cells for contrast genotype × age for mice of the following groups WT_P20 (*n* = 6), *Fmr1* KO_P20 (*n* = 7), WT_P32 (*n* = 6), and *Fmr1* KO_P32 (*n* = 6).

Contrast	Estimate	SE	DF	*t*	Adjusted *p*	*p* summary
WT_P20—WT_P32	−6.00	3.71	21	−1.62	0.38	ns
WT_P20—*Fmr1* KO_P20	−11.10	3.57	21	−3.11	0.02	*
WT_P20—*Fmr1* KO_P32	−8.83	3.71	21	−2.38	0.11	ns
WT_P32—*Fmr1* KO_P20	−5.10	3.57	21	−1.43	0.49	ns
WT_P32—*Fmr1* KO_P32	−2.83	3.71	21	−0.76	0.86	ns
*Fmr1* KO_P20—*Fmr1* KO_P32	2.26	3.57	21	0.63	0.92	ns
WT_P20—WT_P32	−6.00	3.71	21	−1.62	0.59	ns

*Note:* This table is associated with data in Figure 8B. ART‐C Tukey's multiple comparisons test, *p* values, *p < 0.05; ns, not significant.

Taken together, these results suggest that *Fmr1* mutation was associated with a (prematurely) increased number of activated cells in the IC after sound exposure, especially during early auditory development.

### 3.6 AGS Severity Is Correlated With Stronger Neural Synchronicity in Subcortical Auditory Generators

**FIGURE 9:**
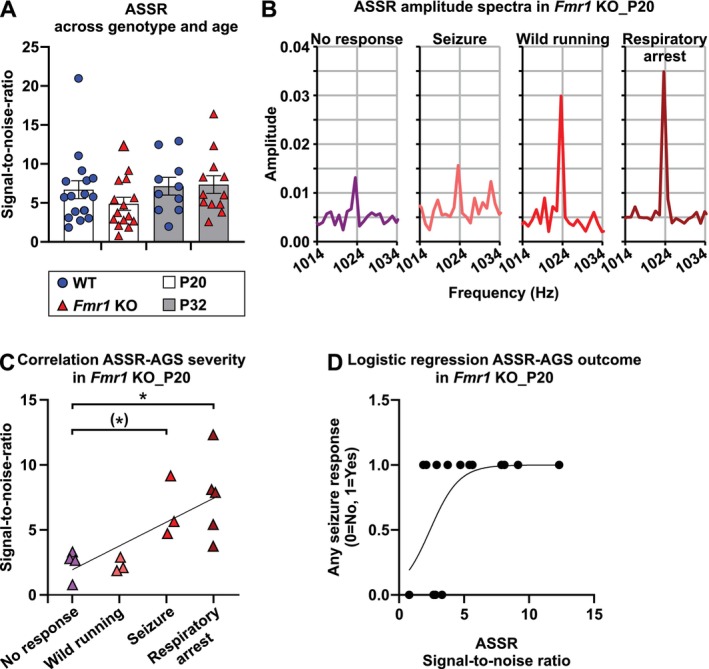
AGS severity is correlated with ASSR signal‐to‐noise ratio amplitude. (A) ASSR signal‐to‐noise ratios from all recorded animals across genotypes and ages: Infant (white bar) and juvenile (gray bar) WT (blue circles) and *Fmr1* KO mice (red triangles) as a measure for neuronal synchronization to an external oscillator (i.e., modulated tone). ASSR signal‐to‐noise ratios were not different between WT_P20 (6.7 ± 1.1), *Fmr1* KO_P20 (4.9 ± 0.8), WT_P32 (7.1 ± 1.1), and *Fmr1* KO_P32 (7.4 ± 1.1) mice. (B) ASSR amplitude spectra, pooled and averaged for *Fmr1* KO_P20 mice depending on their AGS severity: No response (purple line), wild running (light red line), tonic–clonic seizure (red line), respiratory arrest (dark red line). ASSR magnitudes at the first harmonic (1024 Hz) of the modulation frequency increased in *Fmr1* KO_P20 mice with AGS severity. (C) ASSR signal‐to‐noise ratios in the subset of *Fmr1* KO_P20 mice, grouped by AGS phenotype (no response, wild running, seizure, respiratory arrest). In infant *Fmr1* KO mice pooled by AGS phenotype, ASSR signal‐to‐noise ratios were significantly different (1‐way ANOVA *p* = 0.01, *F* (3, 11) = 5.57), in particular between mice with no response and respiratory arrest, and a statistical trend in mice with tonic–clonic seizures. There was a strong positive, statistically significant, correlation between ASSR signal‐to‐noise ratio and AGS severity (Spearman r 0.7713, two‐tailed *p* = 0.001). (D) Binary logistic regression for ASSR signal‐to‐noise ratio and AGS outcome grouped into no response (“Any seizure response [0 = No]”) and any AGS response including wild running, seizure, and respiratory arrest (“Any seizure response [1 = Yes]”). There was a statistically significant relationship between the ASSR signal‐to‐noise ratio and AGS test outcome (Likelihood ratio test *p* = 0.0179, Tjur's R squared 0.3064, curve equation log odds = −2.145 + 0.8983 × *X*). (A) WT_P20 (*n* = 16), *Fmr1* KO_P20 (*n* = 15), WT_P32 (*n* = 10), *Fmr1* KO_P32 (*n* = 12), (B, C) *Fmr1* KO_P20 no response (*n* = 4), KO_P20 wild running (*n* = 3), KO_P20 seizure (*n* = 3), KO_P20 respiratory arrest (*n* = 5), (D) “Any seizure response (0 = No)” (*n* = 4), “Any seizure response (1 = Yes)” (*n* = 11). Data expressed as (A) mean (bars) ± SEM (error bars) and individual animals (symbols), (B) mean (lines), (C) individual animals (symbols) and linear regression line for visual clarity, or (D) individual animals (symbols) and binary simple logistic regression curve. *p* values, (*)*p* < 0.1, *p < 0.05.

Epileptic seizures are caused by stereotyped changes in neurological function leading to hyper‐synchronization of underlying neural networks (Medeiros and Moraes 2014). It has been proposed that the brain pathophysiology associated with seizure susceptibility would compromise the functional synchronization of neural circuitry, even outside of the seizure state (Uhlhaas and Singer 2006; Pinto et al. 2019), and that how easily networks synchronize to an external oscillator may predict brain state changes that lead to seizures (Pinto et al. 2017). In the auditory system, periodic oscillatory activity of neuron ensembles can be externally driven using sinusoidally amplitude‐modulated sounds and measured as auditory steady‐state response (ASSR) electrical potentials. ASSRs are thought to reflect the synchronous discharge of auditory neurons phase‐locked to the modulation frequency of tonal stimulation (Kuwada et al. 2002; Picton et al. 2003; Parthasarathy and Bartlett 2012). To evaluate the synchronous neural activity of subcortical auditory structures, we acoustically stimulated WT_P20, *Fmr1* KO_P20, WT_P32, and *Fmr1* KO_P32 mice with a tone (carrier frequency of 11.3 kHz) modulated in amplitude (512 Hz, Figure 1A) and extracted the signal‐to‐noise ratio (maximum amplitude at the first harmonic of the modulation frequency divided by the noise floor amplitude) of the ASSR spectral magnitude (Figure 1D). Surprisingly, our initial analysis comparing ASSR signal‐to‐noise ratios across genotypes and ages found no statistically significant main effects or interactions of genotype, sex, or age (Figure 9A, Table 17). Such grouping by genotype and age alone might mask potential associations between ASSRs and AGS susceptibility. Therefore, to assess a possible contribution of increased synchronous subcortical activity to AGS susceptibility, we focused on the *Fmr1* KO_P20 subgroups and divided mice by AGS phenotype. We pooled the ASSRs of *Fmr1* KO_P20 mice into the four groups based on AGS phenotypes, that is, *Fmr1* KO_P20 with no response, wild running, tonic–clonic seizure, or respiratory arrest. Averaged ASSR amplitude spectra for each AGS phenotype (Figure 9B) showed a progressive increase in response amplitude at 1024 Hz, suggesting that higher AGS severity is associated with stronger neuronal synchronization. Further subgroup analysis revealed a significant difference in ASSR signal‐to‐noise ratio between these four groups (one‐way ANOVA, *p* = 0.01, *F*(3, 11) = 5.57, Figure 9C). When compared to the *Fmr1* KO_P20 mice with no response as control (mean ± SEM, 2.4 ± 0.6), ASSR signal‐to‐noise ratios were increased significantly in mice with the most severe AGS phenotype (respiratory arrest, 7.5 ± 1.5, Figure 9C, Table 18), and by a statistical trend in mice with tonic–clonic seizures (6.5 ± 1.4, Figure 9C, Table 18), whereas those from mice with wild running were similar (2.3 ± 0.3, Figure 9C, Table 18). Furthermore, there was a strong positive, statistically significant, correlation between the AGS phenotypes and ASSR signal‐to‐noise ratios (Figure 9C, Spearman r 0.7713, two‐tailed *p* = 0.001). In order to assess how well the ASSR signal‐to‐noise ratio predicted the AGS phenotype, we conducted a binary logistic regression test (ASSR signal‐to‐noise ratio versus “Any seizure response (0 = No/1 = Yes)”). We found a statistically significant relationship between the ASSR signal‐to‐noise ratio and AGS test outcome (Figure 9D, Likelihood ratio test *p* = 0.0179, Tjur's R squared 0.3064), where the probability of any seizure response (wild running, tonic–clonic seizure, and respiratory arrest) is 50% at an ASSR signal‐to‐noise ratio value of 2.39 and 75% at an ASSR signal‐to‐noise ratio value of 3.61. In conclusion, these findings suggest that increased phase‐locking and synchronization in the subcortical auditory processing circuits assessed through ASSRs might be associated with the predisposition to more intense AGS severity in *Fmr1* KO mice during early auditory development.

**TABLE 17:** Statistical comparisons of ASSR signal‐to‐noise ratio from mice of the following four groups: WT_P20 (*n* = 16), *Fmr1* KO_P20 (*n* = 15), WT_P32 (*n* = 10), and *Fmr1* KO_P32 (*n* = 12).

Parameter	Source of variation	*p*	*p* summary	*F*(DFn, DFd)	*η*_p_^2^
ASSR	Genotype	0.37	ns	*F*(1, 45) = 0.78	0.017
	Sex	0.77	ns	*F*(1, 45) = 0.08	0.002
	Age	0.14	ns	*F*(1, 45) = 2.24	0.047
	Genotype:Sex	0.22	ns	*F*(1, 45) = 1.51	0.033
	Genotype:Age	0.37	ns	*F*(1, 45) = 0.80	0.018
	Sex:Age	0.67	ns	*F*(1, 45) = 0.18	0.004
	Genotype:Sex:Age	0.35	ns	*F*(1, 45) = 0.86	0.019

*Note:* This table is associated with data in Figure 9A. 3‐way ART ANOVA, *p* values, ns, not significant.

**TABLE 18:** *Post hoc* pairwise comparisons of ASSR signal‐to‐noise ratio from *Fmr1* KO_P20 mice of the following four AGS categories: No response (*n* = 4), wild running (*n* = 3), seizure (*n* = 3), or respiratory arrest (*n* = 5).

Parameter	Contrast	Mean diff.	95% CI of diff.	DF	*q*	Adjusted *p*	*p* summary
ASSR	No response vs. wild running	0.09554	−4.660 to 4.851	11	0.05	> 0.9	ns
	No response vs. seizure	−4.126	−8.881 to 0.6294	11	2.36	0.09	ns
	No response vs. respiratory arrest	−5.113	−9.289 to −0.9359	11	3.33	0.01	*

*Note:* This table is associated with data in Figure 9C. Dunnett's multiple comparisons test, *p* values, *p < 0.05; ns, not significant.

### 3.7 Conclusion

**FIGURE 10:**
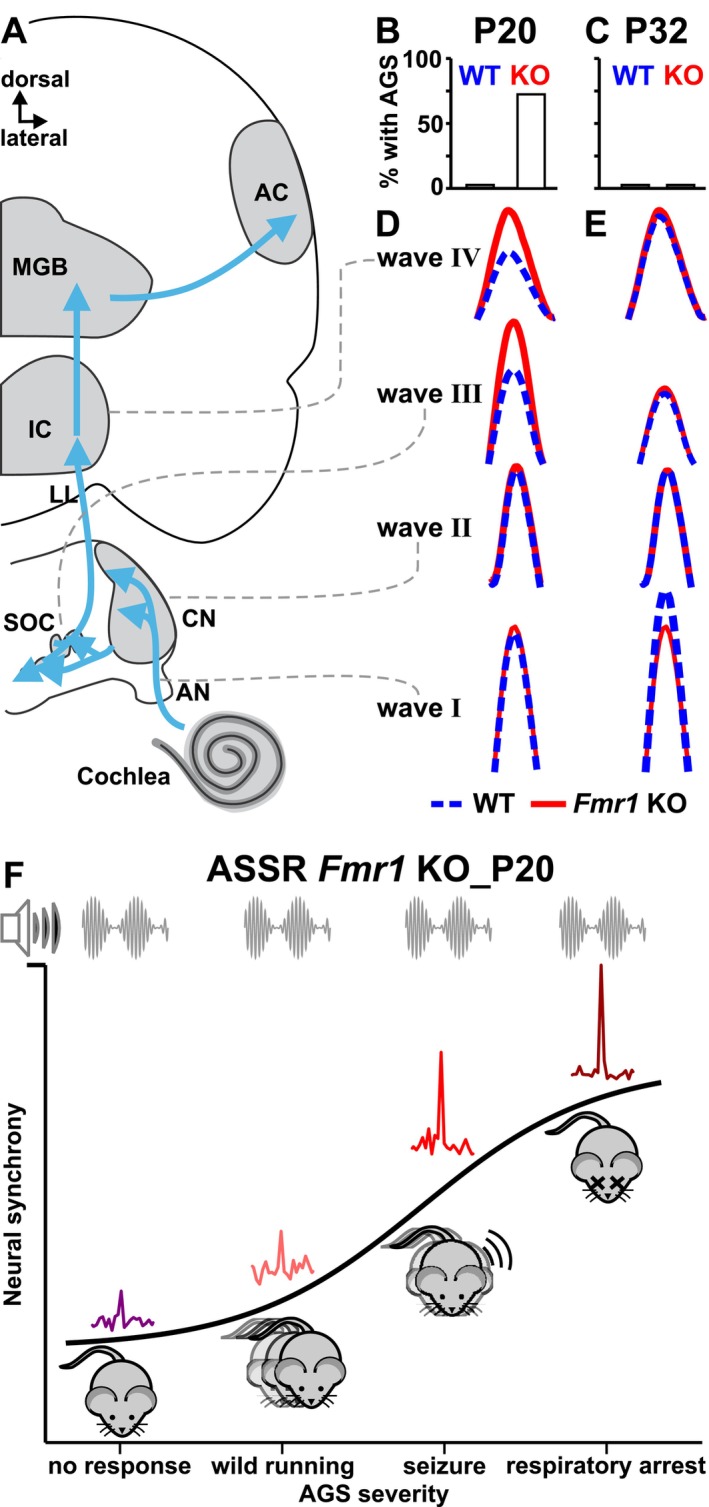
Summary of AGS susceptibility and ABRs in infant (P20) and juvenile (P32) WT and *Fmr1* KO mice, and of ASSRs in the four AGS phenotypes of *Fmr1* KO_P20 mice. (A) Schematic drawing of the auditory pathway and correlated stimulus‐evoked deflections of ABR waves, I, II, III, and IV. The auditory signal along the auditory pathway measured by ABRs provides information regarding auditory function and hearing sensitivity. The different peaks of the ABR wave can be assigned to distinct parts of the ascending auditory pathway: Wave I is generated by the auditory nerve, wave II by the cochlear nucleus, wave III by the SOC, and wave IV by the LL and IC. (B) At P20, AGS incidence was 73% in *Fmr1* KO_P20 mice (including wild running, seizure, and respiratory arrest), and 0% in WT_P20 mice. (C) At P32, AGS susceptibility was 0% in both genotypes. (D) *Fmr1* KO_P20 mice (red solid line) had increased ABR wave III and IV amplitudes compared with WT_P20 mice (blue dashed line). In contrast to infants, (E) ABR wave III and IV amplitudes were similar in *Fmr1* KO_P32 mice (red solid line) and WT_P32 mice (blue line). ABR wave I amplitudes remained lower in *Fmr1* KO_P32 than in WT_P32 mice. (F) Schematic representation of increasing neural synchrony (e.g., ASSR amplitude at the first harmonic of the modulation frequency) across AGS phenotypes in *Fmr1* KO_P20 mice. Neural responses become progressively more synchronized to the amplitude‐modulated auditory stimulus (gray) with increasing AGS severity, as indicated by elevated ASSR amplitude. AC, auditory cortex; AN, auditory nerve; CN, cochlear nucleus; IC, inferior colliculus; LL, lateral lemniscus; MGB, medial geniculate body; SOC, superior olivary complex.

This study highlights the contribution of the *Fmr1* mutation to altered auditory maturation and sound responsiveness in distinct parts of the auditory pathway, during a time period that coincides with a critical window for auditory development. At the beginning of this window, that is, in P20 mice, the *Fmr1* mutation led to increased ABR wave III and IV amplitudes, generated by the SOC or LL and IC, respectively (Figure 10A,D). The hyperresponsiveness at the level of the SOC in the brainstem appeared to be relayed to the IC in the midbrain where it was then amplified, likely facilitating these animals' susceptibility to AGS in response to loud sounds (Figure 10B). Towards the end of the critical window, that is, by P32, ABR wave III and IV amplitudes had stabilized at values similar to those of WT mice (Figure 10E), and AGS responses had ceased (Figure 10C). Taken together, this suggests that the proper maturation of ABR waveforms, reflecting the propagating sound response along the auditory pathway, was disturbed early during this critical time window, likely contributing to FXS‐like sound hypersensitivity. Furthermore, at P20, the ASSRs to amplitude‐modulated stimuli, which assess periodic electrical oscillations in the subcortical auditory regions, increased in *Fmr1* KO mice as the severity of their AGS phenotype increased (Figure 10F). This points to the notion that hyper‐synchronization of subcortical auditory neuron ensembles may be linked to increased proneness to sound hypersensitivity.

In summary, our findings indicate that higher neural responsiveness to sound, as well as hyper‐synchronization to a continuous modulated sound, promotes susceptibility to and severity of AGS in infant *Fmr1* KO mice.

## 4 Discussion

In this study, we report that *Fmr1* mutation leads to characteristic changes in sound processing from auditory periphery to midbrain, likely due to altered maturation of the auditory system during early postnatal development. These changes are most prominent during infancy, suggesting that a transient impairment in auditory processing underlies a heightened susceptibility to AGS in the *Fmr1* KO mouse. Our findings complement previous reports of prolonged AGS developmental trajectories (Musumeci et al. 2000; Yan et al. 2005) by showing that a lower‐intensity stimulus (i.e., 90 dB SPL) can selectively induce AGS in *Fmr1* KO mice during infancy, highlighting the importance of stimulus parameters in targeting specific vulnerable windows.

Our work confirms prior findings that hyperexcitability in the IC is critical for AGS susceptibility in *Fmr1* KO mice (Gonzalez et al. 2019; Nguyen et al. 2020). We refine the link between electrophysiological signals and AGS susceptibility through stratifying ABR data not only by genotype but also by AGS phenotype, revealing a strong correlation between ABR wave III and IV amplitude growth slopes that highlights the SOC as a candidate lower brainstem nucleus that may contribute to pathological hyperresponsiveness. Finally, we report for the first time that alterations in the first harmonic of the ASSR, a measure previously implicated in neuropsychiatric disorders (Gautam et al. 2023; Swerdlow et al. 2024), might serve as a predictive marker for auditory hypersensitivity in neurodevelopmental conditions such as FXS.

Notably, our study includes both male and female mice to characterize age‐related progression of auditory processing deficits associated with AGS susceptibility, addressing a knowledge gap as prior studies have predominantly focused on males (Bartholomay et al. 2019; Kat et al. 2022). Together, these novel insights broaden our understanding of the neurodevelopmental trajectory of auditory dysfunction in FXS and underscore the potential utility of electrophysiological signatures underlying auditory hypersensitivity in FXS.

### 4.1 Possible Contribution of Peripheral Auditory Maturation to Central Mechanisms of AGS Susceptibility

The auditory periphery comprises the cochlear sensory epithelium and the auditory nerve, structures that can be functionally assessed through ABR thresholds and ABR wave I. Auditory thresholds are known to provide a sensitive measure of outer hair cell function, which enhance hearing sensitivity via cochlear amplification (Liberman and Kiang 1978; Liberman and Beil 1979; Dallos and Evans 1995; Dallos 2008). The maturation of the cochlea follows what is known as the “sliding place principle” (Rubel et al. 1984), that is, from middle‐to‐apical and middle‐to‐basal cochlear regions. Accordingly, the developmental increase in hearing sensitivity is frequency‐dependent (Ehret 1976). In the present study, we found WT‐like ABR thresholds in *Fmr1* KO mice at P20 for 4 kHz and 11 kHz (Figure 3A), indicating relatively normal maturation in apical and medial cochlear regions by that age. However, thresholds were elevated at 32 kHz (Figure 3A), suggesting a delay in basal cochlear maturation. This aligns with previous findings that FMRP is expressed in cochlear hair cells during the prehearing period (~P12, Mikaelian and Ruben 1965), but rapidly downregulated during maturation, with no tonotopic differences (Wang, Fan, et al. 2023). These data, along with WT‐like ABR thresholds in juvenile (Figure 3A) and young adult *Fmr1* KO mice (Ferraguto et al. 2023), suggest a role for FMRP in hearing onset and timely cochlear development (Yu and Wang 2023), but not in maintaining mature auditory thresholds. Outer hair cell function also appears preserved into adulthood, as distortion product otoacoustic emissions remain unaltered in *Fmr1* KO mice at 3 months (Ferraguto et al. 2023). Taken together, this supports a model in which cochlear maturation in the absence of FMRP follows a typical tonotopic pattern but is developmentally delayed, stabilizing to WT‐like thresholds by the juvenile stage.

In contrast, FMRP in spiral ganglion neurons, that is, cell bodies of auditory nerve fibers, increases postnatally and remains elevated into adulthood (Wang, Fan, et al. 2023). Although ABR wave I amplitudes, reflecting auditory nerve activity, are generally unchanged in early development (P15, El‐Hassar et al. 2019; P20, Figure 6C of the present study), they are reduced in *Fmr1* KO mice by P32 (Figure 6D) and in adulthood (Rotschafer et al. 2015; Ferraguto et al. 2023). This reduction has been linked to cochlear deafferentation, with loss of ribbon synapses of inner hair cells in the medial cochlea (Ferraguto et al. 2023), known to impair synchronous sound‐evoked activation of spiral ganglion neurons (Khimich et al. 2005). FMRP may play a role in regulating α‐amino‐3‐hydroxy‐5‐methyl‐4‐isoxazole propionic acid receptors (AMPAR) subunit composition at ribbon synapses (Raman et al. 1994; Gardner et al. 1999; Ruel et al. 1999; Glowatzki and Fuchs 2002), based on evidence from brain tissues showing disrupted trafficking of AMPAR subunits GluA2, GluA3, and GluA4 in the absence of FMRP (Chojnacka et al. 2023). GluA3 KO mice similarly show pathological alterations in cochlear synapse structure and function that precede reductions in wave I amplitude in young adults (García‐Hernández et al. 2017; Rutherford et al. 2023). These findings raise the possibility that altered AMPAR subunit composition and trafficking at the cochlear ribbon synapses may disrupt maturation of auditory synaptic transmission, contributing to early pathological changes that precede auditory nerve dysfunction in juvenile *Fmr1* KO mice.

Altered development of the auditory periphery is likely to have secondary effects on central auditory maturation and processing (Rubel and Fritzsch 2002; Polley et al. 2013; Ryugo 2015; Lesicko and Llano 2017). Selective deletion of FMRP in spiral ganglion neurons delays closure of a critical period in the ventral cochlear nucleus, similar to constitutive *Fmr1* KO mice (Yu and Wang 2023). This delay temporally coincides with reduced hearing sensitivity, suggesting an association between auditory brain development and sensory inputs shaped by peripheral FMRP (Yu and Wang 2023). It has been suggested that developmental deficits in acoustic input might lead to compensatory gain in the auditory brainstem, potentially contributing to AGS susceptibility (Faingold et al. 1990). In our study, delayed maturation of hearing sensitivity was associated with increased AGS susceptibility, as mice exhibiting seizure phenotypes (wild running, tonic–clonic seizure, respiratory arrest) had elevated ABR thresholds compared to WT controls at 32 kHz (Figure 3B). However, ABR wave I amplitudes were not similarly associated with AGS susceptibility (11.3 kHz: Figures 6 and 7; 4 and 32 kHz: Table S10). This suggests that, while aspects of peripheral auditory development may relate to AGS susceptibility, altered auditory nerve output alone does not account for AGS. Together with prior work in the mouse model (Garcia‐Pino et al. 2017; El‐Hassar et al. 2019) and humans (St Clair et al. 1987; Arinami et al. 1988; Ferri 1989; Castrén et al. 2003; Van der Molen et al. 2012) our findings support the notion that auditory hypersensitivity in FXS is due to central auditory dysfunction, potentially shaped or exacerbated by atypical early peripheral development. Future studies should investigate to what extent correcting the maturation of the auditory periphery can rectify central auditory function and hypersensitivity in *Fmr1* KO mice.

### 4.2 Correlation Between Altered Maturation of the Auditory Brainstem and AGS Susceptibility

In patients with FXS, auditory processing impairment is predominantly evident in the prolonged latencies of ABR waves III and V (equivalent to rodent wave IV) and in the interpeak intervals III‐V and I‐V (Arinami et al. 1988). Consistent with these findings, the increased amplitudes of ABR waves III (Figure 6C) and IV (P15, El‐Hassar et al. 2019; P20, Figure 6C of the present study) in infant *Fmr1* KO mice suggest heightened excitability in these brainstem nuclei as the neuronal basis underlying auditory hypersensitivity during early development. These differences were absent in the juvenile stage (waves II, III, and IV, Figure 6D) and adulthood (12 kHz, Rotschafer et al. 2015), when AGS susceptibility had subsided. In contrast, ABR peak latencies remained largely unchanged in *Fmr1* KO mice across development (P15, waveI and IV, El‐Hassar et al. 2019; P20 and P32, wave I‐IV, Figure 4) and adulthood (Rotschafer et al. 2015; Chawla and McCullagh 2022), possibly due to species‐specific processing differences or the compact structure of the mouse auditory brainstem (Rotschafer et al. 2015).

We found that the slopes of ABR amplitude growth functions increased with AGS susceptibility, particularly at later peaks attributed to the LL and IC (Figure 7A), indicating enhanced neural response sensitivity to sound intensity. This aligns with previous single unit recordings, showing broader frequency tuning and increased neural responsiveness of IC neurons in *Fmr1* KO mice during development, suggesting that more IC neurons respond synchronously to sounds (Rotschafer and Razak 2013; Nguyen et al. 2020). Immunohistochemical labeling of cFos further showed increased density of IC cell activation in *Fmr1* KO mice in the dorsolateral IC at P21, shifting to the ventromedial IC by P34 (Nguyen et al. 2020). To avoid triggering AGS and minimize confounds from locomotor activity (Yang et al. 2020), Nguyen et al. (2020) used 85 dB SPL for infants and 80–90 dB SPL for juveniles. In contrast, our study used a consistent 90 dB SPL across all groups and found no age‐related effects on cFos‐positive cell density throughout the IC's dorso‐ventral axis (Figure 8). In our study, the genotype effect was primarily driven by the increased number of cFos‐positive cells in *Fmr1* KO mice at P20 compared to WT controls (Figure 8). Differences in sound frequency range and modulation between our study and Nguyen et al. (2020) may account for the slight discrepancies in cFos cell counts. We further did not observe a systematic influence of the AGS phenotype on cFos labeling, though a higher sample size is needed to confirm this. Interestingly, there was no evidence for abnormal development of IC frequency tonotopy (Nguyen et al. 2020), matching our respective conclusion on the auditory periphery. Overall, our findings suggest that auditory hyperresponsiveness during early development may originate in lower brainstem structures such as the SOC and become pathologically amplified in the IC.

Supporting this, Gonzalez et al. (2019) showed that *Fmr1* deletion in IC glutamatergic neurons is necessary, but not sufficient, to induce AGS, requiring *Fmr1* deletion in other subcortical vGlut2 (vesicular glutamate transporter 2)‐expressing glutamatergic neurons to fully elicit the phenotype (Gonzalez et al. 2019). Among the most likely subcortical sources of vGlut2 terminals in the IC (i.e., IC, LL intermediate nucleus, dorsal cochlear nucleus, medial and lateral superior olive, Blaesse et al. 2005; Ito and Oliver 2010; Ito et al. 2011), the lateral superior olive exhibits prominent structural and functional changes in *Fmr1* KO mice. Between prehearing (P6) and shortly after hearing onset (P14), vGlut2 fractional coverage decreases in both WT and *Fmr1* KO mice (Rotschafer and Cramer 2017); however, *Fmr1* KO mice later show a greater vGlut2‐immunolabeled area within the lateral superior olive (Rotschafer et al. 2015). In line with this morphological difference, synaptic functional maturation in the lateral superior olive is severely altered during the first 10 days after hearing onset in *Fmr1* KO mice, resulting in enhanced ipsilateral excitatory input and an excessive number of excitatory synapses (Garcia‐Pino et al. 2017). Lateral superior olive neurons in these mice exhibit increased firing rates, broadened tuning curves, and shifted interaural level differences (Garcia‐Pino et al. 2017), which are critical for sound localization (Grothe et al. 2010). Additionally, morphological alterations of the SOC nuclei and neurons have been observed in post‐mortem tissue of subjects with FXS and autism (Kulesza and Mangunay 2008; Kulesza et al. 2011).

There is growing evidence that inhibitory signaling is disrupted in *Fmr1* knockout mice, particularly during key developmental windows when auditory circuits mature (Song et al. 2021). FMRP is expressed in GABAergic neurons (Feng et al. 1997; Olmos‐Serrano et al. 2010), and reduced GABA_A_ receptor subunit expression or binding has been reported in both *Fmr1* KO mice (El Idrissi et al. 2005; D'Hulst et al. 2006; Gantois et al. 2006) and humans (D'Hulst et al. 2015). Early in development, GABA acts depolarizing due to high intracellular chloride (Represa and Ben‐Ari 2005); in *Fmr1* KO mice, the switch to hyperpolarizing GABA is delayed in cortex and hippocampus (He et al. 2014; Tyzio et al. 2014), potentially affecting inhibitory circuit maturation and excitation/inhibition balance in the auditory brainstem. In the IC, increasing inhibitory tone between P14 and P21 sharpens frequency tuning and reduces response magnitude in WT mice, but seems impaired in *Fmr1* KO mice, possibly leading to broader tuning and hyper‐responsiveness related to auditory hypersensitivity (Grimsley et al. 2013; Sturm et al. 2017; Nguyen et al. 2020). In the lower brainstem, *Fmr1* KO rodent models show fewer GABAergic neurons in the superior paraolivary nucleus (Ruby et al. 2015) and decreased glycinergic and GABAergic inhibition to the medial nucleus of the trapezoid body indicated by GAD67 and GlyT2 expression, with no change in glycinergic or GABAergic presynaptic structures in the lateral superior olive (McCullagh et al. 2017).

While our data did not reveal significant AGS phenotype‐related changes in the slopes of ABR wave III growth functions (Figure 7A), we found a strong correlation between the slopes of waves III and IV (Figure 7B), further implicating ascending brainstem structures, especially the SOC and IC, in the amplification of auditory hyperexcitability. Looking forward, broader histological and physiological assessments of lower brainstem circuits are warranted to better resolve the cellular mechanisms underlying seizure susceptibility in *Fmr1* KO mice, in particular when stratified for AGS phenotype and effects of sex. These should include markers for neuronal activity (e.g., cFos), and excitatory (e.g., vGlut2) and inhibitory transmission (e.g., GAD67, GlyT2), as well as imaging techniques like Matrix‐assisted laser desorption/ionization to quantify GABA and glutamate neurotransmitter levels in auditory brainstem nuclei (Möhrle et al. 2021). Finally, clarifying the specific contributions of individual auditory brainstem generators to auditory hypersensitivity is needed. While ABRs are widely used in preclinical models, with waves I and II commonly attributed to the auditory nerve and cochlear nucleus (for overview see Wang et al. 2025), the origins of later waves in mice remain more debated. Recent evidence by Wang et al. (2025) and Land et al. (2016) supports a prominent role of the IC in wave IV, while the subsequent wave V is likely superimposed on higher‐order activity. In this context, in vivo unit recordings across development in *Fmr1* KO mice may help pinpoint alterations in response magnitude, threshold, and duration in specific brainstem nuclei involved in AGS. Complementary approaches, such as miniature microscopy for deep tissue imaging, may further reveal single‐cell or population‐level neural activity in behaving mice. Together, these investigations will be critical for identifying the neural substrates and pathological mechanisms underlying auditory hypersensitivity in FXS and autism.

### 4.3 ASSR Amplitudes Might Be Useful to Predict AGS Severity

We found no differences in ASSR signal‐to‐noise ratios between WT and *Fmr1* KO mice at P20 and P32 (Figure 9A), indicating preserved phase‐locking to amplitude‐modulated tones in subcortical auditory neurons. This is consistent with Nguyen et al. (2020), who found no genotypic differences in single neuron phase locking in the IC across comparable developmental stages (P14, P21, P34). ASSRs are known to mature with age, as reflected by increasing envelope following response and shifts in the modulation frequency range of maximal responsiveness in rats (Prado‐Gutierrez et al. 2012; Venkataraman and Bartlett 2014) and humans (Pethe et al. 2004; Savio et al. 2001; Mijares Nodarse et al. 2012). It is possible that age‐related and/or genotypic differences would emerge under different combinations of carrier and modulation frequencies than those tested here.

While genotype alone did not influence ASSR signal‐to‐noise ratios, we observed a robust association between ASSR amplitudes and AGS severity in *Fmr1* KO mice (no response, wild running, seizure, or respiratory arrest). ASSR signal‐to‐noise ratios positively correlated with AGS phenotype (Figure 9C) and significantly predicted AGS outcomes at P20 (Figure 9D). These findings suggest that higher ASSR amplitudes may reflect increased neural network susceptibility to hypersynchronous activity, which contributes to AGS. Supporting this, Wistar audiogenic rats show increased ASSR‐normalized energy at sub‐AGS sound intensities (85 dB SPL, Pinto et al. 2017), suggesting that elevated ASSR responses may precede seizure susceptibility. ASSRs have also been proposed as a neural correlate of perceived loudness in humans (e.g., inaudible to unbearable, Van Eeckhoutte et al. 2016), speaking to the usefulness of ASSR amplitudes as an electrophysiological correlate to estimate the loudness percept and possibly auditory hypersensitivity. Future work is needed to determine the mechanisms underlying the increased ASSR amplitudes in *Fmr1* KO mice with higher AGS severity. Developmental factors such as myelination, synaptic maturation, and changes in membrane properties likely contribute to enhanced phase‐locking capacity during the weeks following hearing onset (Scott et al. 2005; Ryugo et al. 2006; Prado‐Gutierrez et al. 2012). An additional consideration is stress sensitivity. In the absence of FMRP, stress induces more immediate corticosteroid responses (Ghilan et al. 2015), which might in turn affect ASSR magnitudes (Marchetta et al. 2022) and AGS severity in subsets of *Fmr1* KO mice that are more vulnerable to stress.

The neural generators dominating the ASSR signal depend on the modulation frequency. Higher frequencies (~1000 Hz) reflect early neural sources including the auditory nerve (Palmer and Evans 1982; Joris and Yin 1992; Shaheen et al. 2015) and cochlear nucleus (Joris et al. 2004), while responses to 200–500 Hz are primarily generated in the brainstem and midbrain (Kiren et al. 1994; Kuwada et al. 2002; Picton et al. 2003; Parthasarathy and Bartlett 2012). In conclusion, while ASSRs were not altered by genotype, their relationship to AGS severity in *Fmr1* KO mice suggests that they may serve as a useful, diagnostic tool for assessing altered neural synchronization in distinct neuronal generators that contribute to auditory hypersensitivity in neurodevelopmental disorders.

### 4.4 Methodological Considerations, Limitations, and Translational Potential

While *Fmr1* KO mice have advanced our understanding of FXS, they do not fully replicate the human condition. In humans, full *FMR1* mutations (> 200 CGG repeats) cause promoter hypermethylation and gene silencing (Oberlé et al. 1991; Verkerk et al. 1991; Hagerman et al. 2001), whereas *Fmr1* KO mice lack FMRP due to *Fmr1* gene disruption (Bakker et al. 1994; Yan et al. 2004). Such mechanistic differences may explain why some clinical features (e.g., cognitive impairments, anxiety) are inconsistently modeled (reviewed in e.g., Kazdoba et al. 2014; Kat et al. 2022; Willemsen and Kooy 2023). Yet, both species show overlapping auditory processing deficits, including hypersensitivity, reduced sound tolerance, and impaired sensorimotor gating (Frankland et al. 2004; Sinclair et al. 2017; Rais et al. 2018; Salvi et al. 2022), linked to hyperexcitability from FMRP deficiency (e.g., altered auditory evoked brain potentials, electroencephalograms, and neural synchrony, Musumeci et al. 2000; Berry‐Kravis 2002; Castrén et al. 2003; Lozano et al. 2014; Ethridge et al. 2017; Lovelace et al. 2018). Seizure susceptibility in FXS and *Fmr1* KO mice peaks early in development. Childhood epilepsy affects 10%–20%, often remitting by adolescence (Musumeci et al. 1999; Berry‐Kravis 2002; Kaufmann et al. 2017), though some adults continue to experience seizures (Incorpora et al. 2002; Gauthey et al. 2010; Stone et al. 2023). Similarly, *Fmr1* KO mice show heightened AGS susceptibility in early development (e.g., Berry‐Kravis 2002; Yan et al. 2005; Willemsen and Kooy 2023). Spontaneous seizures are rare but reported in *Fmr1* KO mice on the FVB background during adulthood (~P100) depending on developmental noise environment (Armstrong et al. 2022). This supports the notion that fundamental sensory circuits and hyperexcitability are conserved across species, with auditory experiences during critical periods shaping network excitability (Yan et al. 2005; Kulinich et al. 2020; Armstrong et al. 2022).

In our study, *Fmr1* KO mice showed robust AGS susceptibility at P20 but none by P32 (Figure 2). The 73% AGS incidence at P20 matches prior reports (~60%–100%, e.g., Musumeci et al. 2000; Dölen et al. 2007; Osterweil et al. 2010; Muscas et al. 2019). However, many studies with *Fmr1* KO mice on the FVB background show AGS susceptibility persisting into juvenile (P29–35, Musumeci et al. 2000; Yan et al. 2004; Yan et al. 2005; Min et al. 2009) and adult stages (> P60, Yan et al. 2004; Qin et al. 2005; Yan et al. 2005; Armstrong et al. 2020). In comparison, *Fmr1* KO mice on the C57BL/6J background demonstrate a narrower window declining by P48 (Yan et al. 2005). In some studies, the FVB WT controls also showed substantial AGS incidence (~20%–30%, Musumeci et al. 2000; Yan et al. 2004; Yan et al. 2005; Min et al. 2009), pointing to the heightened sensitivity of the FVB strain to AGS. In contrast, in both our study and reports by Chen and Toth (2001), FVB WT controls did not exhibit AGS at any age, and adolescent *Fmr1* KO mice between P42–48 similarly showed no AGS (Chen and Toth 2001). Differences in AGS susceptibility across studies likely reflect stimulus variations: our 90 dB SPL siren and the AGS sound in the Chen and Toth (2001) study (115 dB SPL) were lower than commonly used 120–125 dB SPL levels, with differences in duration and frequency range as well (5 min, 4.8 mean and 12 Hz amplitude modulation frequency, our study; 1 min, 2–10 kHz, Chen and Toth 2001; 15 min, 1.8–6.3 kHz, Yan et al. 2005). In line with this, Auerbach et al. (2021) showed that loudness perception in *Fmr1* KO rats is influenced differently from WTs by sound intensity, frequency, and bandwidth, suggesting stimulus parameters critically impact phenotype expression. Importantly, the 90 dB SPL AGS siren intensity matched the maximum sound level used in our ABR and ASSR protocols and falls within a physiological sound intensity range akin to environmental noise (Sygrove 2024). The heightened AGS sensitivity of the FVB strain made *Fmr1* KO mice on this genetic background especially well‐suited for detecting AGS at an intensity well below the pain threshold. This may be particularly relevant given that individuals with auditory hypersensitivity commonly report moderate intensity sounds as unbearably loud (Gomes et al. 2008; Rotschafer and Razak 2014; McCullagh et al. 2020). Though likely not maximizing seizure incidence, this intensity allowed us to selectively induce AGS in *Fmr1* KO P20 mice and assess associated auditory physiology within a transient developmental window.

As reviewed by Razak et al. (2020), FXS phenotypes, including auditory processing abnormalities, evolve across development, with shifts in phenotype expression from infancy through juvenile and adult stages (e.g., Yun et al. 2006; Gauducheau et al. 2017; Rotschafer and Cramer 2017; Wen et al. 2019). Our study focused on P20 and P32, but did not assess early adulthood (~P60), when persistent or late‐emerging auditory phenotypes may manifest. The absence of AGS at P32 may reflect age‐related changes in circuit excitability and/or the moderate stimulus intensity used. A broader range of acoustic stimuli, such as higher intensities, longer exposures, or different frequency components, may potentially reveal higher AGS incidence, particularly at P32. Future studies should also include later developmental stages (e.g., P60) to fully capture the trajectory of auditory circuit dysfunction and AGS susceptibility in FXS models. Additionally, awake electrophysiological recordings during seizure phases could elucidate the temporal dynamics of auditory processing underlying AGS susceptibility.

The developmental trajectory of AGS severity might be dependent on sex as well. Similar to our results on P20, Musumeci et al. (2000) and Ding et al. (2014) found no differences in AGS severity between male and female *Fmr1* KO mice (FVB background, P22 to 45; C57BL/6 background P21 to 24, respectively). However, females exhibited their peak severity on P22, whereas that of males progressively increased with age (Musumeci et al. 2000). In line with AGS severity, we found no sex differences in hearing function at P20. At P32, minor sex differences were observed: ABR wave III amplitudes in response to 11.3 kHz were lower in females irrespective of genotype (Table 13), and wave I latencies to 32 kHz were prolonged in *Fmr1* KO males compared to females (Figure 5C). Generally speaking, the literature on sex differences in ABR wave characteristics appears inconsistent (Hunter and Willott 1987; Guimaraes et al. 2004; Kim et al. 2023; Lozier et al. 2023). In adult *Fmr1* KO mice, it has been reported that females have lower wave IV amplitudes, while neither sex showed changes in latency or hearing range (> P60, Chawla and McCullagh 2022). In humans, the majority of females with FXS are heterozygous and tend to have milder phenotypes due to X‐chromosome inactivation, which allows partial compensation from the second, unaffected *FMR1* allele (Werling and Geschwind 2013). This gene dosage effect is also evident in certain phenotypes in the animal model. For example, heterozygous female *Fmr1* KO mice show reduced AGS incidence compared to homozygous females or hemizygous males (Musumeci et al. 2000). Sexually dimorphic characteristics in *Fmr1* KO mice have been described for autism‐like behaviors, such as sociability, as well as for aspects of brain development (Wang, Qiao, et al. 2023). Other phenotypes, such as hyperlocomotion, may emerge at a later age or remain sex‐independent (Ding et al. 2014; Gauducheau et al. 2017). These findings emphasize the need to incorporate age and sex as biological variables in auditory research to improve the accuracy and generalizability of results. Future studies are needed to explore the effect of sex on hearing function in *Fmr1* KO mice in greater depth, especially when stratified for AGS severity.

## 5 Conclusion

Taken together, our findings highlight a transient impairment in auditory processing during infancy that may underlie heightened AGS susceptibility in the FXS pathology. The absence of most phenotypic differences by P32, while unexpected in light of previous reports, likely reflect age related‐processing changes in response to the specific auditory stimulus parameters used in this study. As such, our findings may represent a lower‐bound estimate of AGS and auditory hyperresponsiveness. Complementing existing literature, our results suggest that the developmental trajectory of AGS and electrophysiological phenotypes in *Fmr1* KO mice may be highly dependent on the intensity and nature of sensory input, particularly during early sensitive periods.

Our study demonstrates that the *Fmr1* mutation disrupts auditory processing primarily during a transient early developmental window, predominantly within the central auditory system. While delayed maturation of peripheral hearing sensitivity may contribute to AGS susceptibility, auditory nerve output alone does not directly correlate with AGS susceptibility. Rather, our data suggest that altered central processing, especially in the brainstem and midbrain, is the primary driver of AGS vulnerability. These findings underscore the importance of stimulus characteristics and developmental timing in shaping auditory circuit function, and that early developmental interventions targeting auditory processing might offer a critical therapeutic window to alleviate auditory hypersensitivity in FXS.

## Author Contributions

Dorit Möhrle designed the study, performed experiments, MATLAB coding, formal analysis, data interpretation and visualization, wrote the original draft, and contributed to funding. Demi Ma performed experiments, formal analysis, and participated in writing the original draft. Wenyue Xue participated in experiments and editing of the manuscript. Jun Yan provided resources and contributed to editing of the manuscript. Ning Cheng provided funding, contributed to study design and editing of the manuscript. All authors read and approved the final manuscript.

## Funding

This work was supported by Alberta Children's Hospital Research Foundation, Faculty of Veterinary Medicine, University of Calgary, Kids Brain Health Network, Brain Canada, Natural Sciences and Engineering Research Council of Canada, Hotchkiss Brain Institute, Owerko Centre, Alberta Innovates.

## Conflicts of Interest

The authors declare no conflicts of interest.

## Supporting information


**Data S1:** aur70152‐sup‐0001‐Supinfo.pdf.

## Data Availability

The data that support the findings of this study are available from the corresponding author upon reasonable request.
