# Supplementary material for: Altered Auditory Maturation in Fragile X Syndrome and Its Involvement in Audiogenic Seizure Susceptibility
Source: Autism Res. 2025 Dec 15;19(1):e70152. doi: 10.1002/aur.70152 (PMC12853251; doi:10.1002/aur.70152)
Supplement: Supplementary file 1 — Data S1: aur70152‐sup‐0001‐Supinfo.pdf. [file AUR-19-0-s001.pdf]

Supplementary Material

1 Supplementary Figures

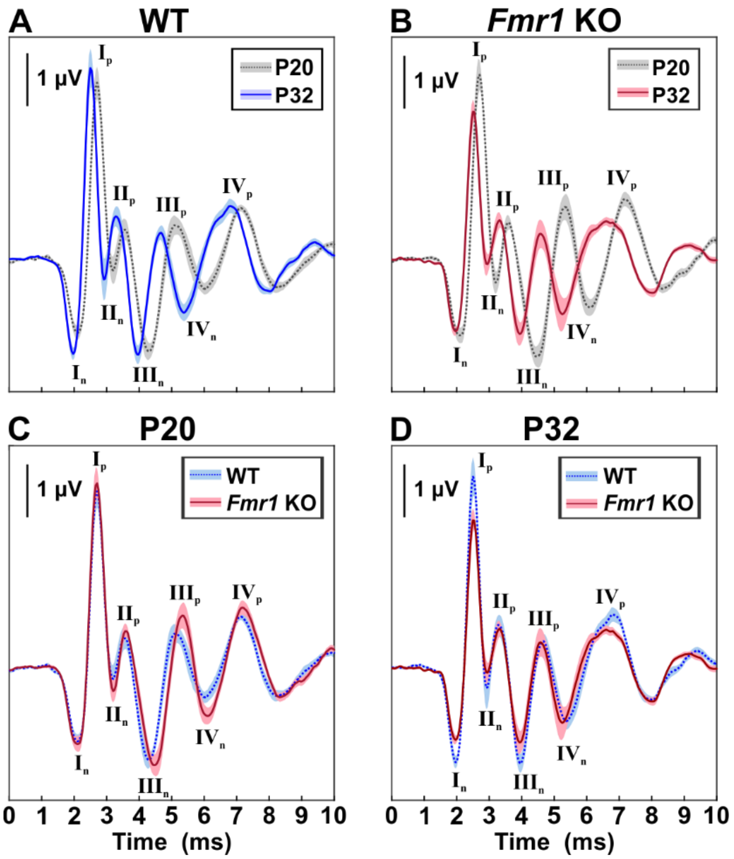

Supplementary Figure 1. ABR waveforms at 60 dB *re* threshold. ABR waveforms consist of consecutive amplitude deflections (waves), with each wave consisting of a starting negative (n) peak and the following positive (p) peak. ABR wave I: I_n-I_p, wave II: II_n-II_p, wave III: III_n-III_p, wave IV: IV_n-IV_p. Wave latencies were defined by the onset timing (negative peak) of each corresponding wave, and wave amplitudes by the peak-to-peak difference. ABR waveforms for **(A)** WT_P20 (gray dotted line and area) and WT_P32 (blue line and area), **(B)** *Fmr1* KO_P20 (gray dotted line and area) and *Fmr1* KO _P32 (red line and area), **(C)** WT_P20 (blue dotted line and area) and *Fmr1* KO_P20 (red line and area), and **(D)** WT_P32 (blue dotted line and area) and *Fmr1* KO_P32 (red line and area). WT_P20 (*n*=16), *Fmr1* KO_P20 (*n*=15), WT_P32 (*n*=10), and *Fmr1* KO_P32 (*n*=12). Data expressed as mean (lines) ± SEM (shaded areas).

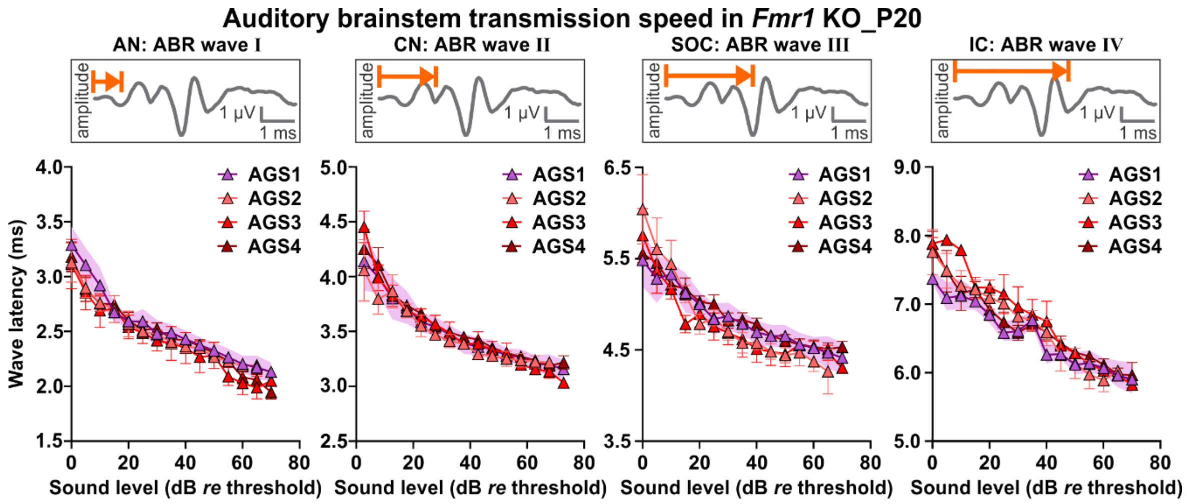

Supplementary Figure 2. ABR wave I to IV negative peak latencies were similar in *Fmr1* KO_P20 mice grouped by AGS phenotype. ABR wave latencies corresponding to auditory nerve (AN, wave I), cochlear nucleus (CN, wave II), superior olivary complex (SOC, wave III), and lateral lemniscus and inferior colliculus (LL and IC, wave IV) in response to pure tone stimuli (11.3 kHz) with increasing sound intensity. Latencies were compared between *Fmr1* KO_P20 with no response (AGS1, purple triangles and line), wild running (AGS2, light red triangles and line), seizure (AGS3, red triangles and line), respiratory arrest (AGS4, dark red triangles and line). Latencies were not significantly different between the four phenotypes for ABR waves I, II, III, and IV (Supplementary Table 9). *Fmr1* KO_P20 AGS1 (*n*=4), AGS2 (*n*=3), AGS3 (*n*=3), AGS4 (*n*=5). Data expressed as mean (symbols and lines) ± SEM (shaded area and error bars).

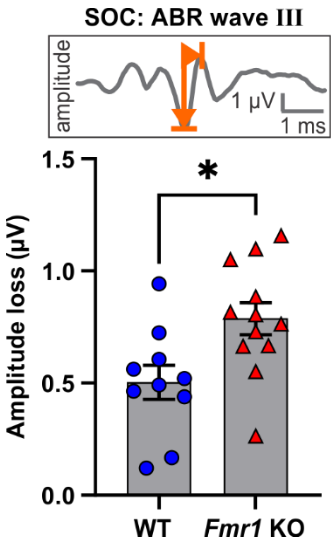

Supplementary Figure 3. Developmental decrease of ABR wave III **amplitudes in WT and *Fmr1* KO mice.** Amplitude loss was significantly greater in *Fmr1* KO mice (red triangles) compared to WT mice (blue circles, unpaired *t* test, $p = 0.013$). To calculate amplitude loss across development, wave III amplitudes were first averaged within each genotype at P20 across sound levels from 15 to 45 dB *re* threshold. These genotype-level P20 averages were then subtracted from the corresponding wave III amplitudes recorded from each P32 animal at the same dB *re* threshold levels. The resulting differences (i.e., amplitude reductions) were averaged across the 15–45 dB *re* threshold range for each P32 animal to yield a single mean amplitude loss value per mouse. Abbreviation: SOC, superior olivary complex WT_P20 (n=16), *Fmr1* KO_P20 (n=15), WT_P32 (n=10), and *Fmr1* KO_P32 (n=12). Data expressed as mean (bars) ± SEM (error bars) and individual animals (symbols). p value, * $p < 0.05$.

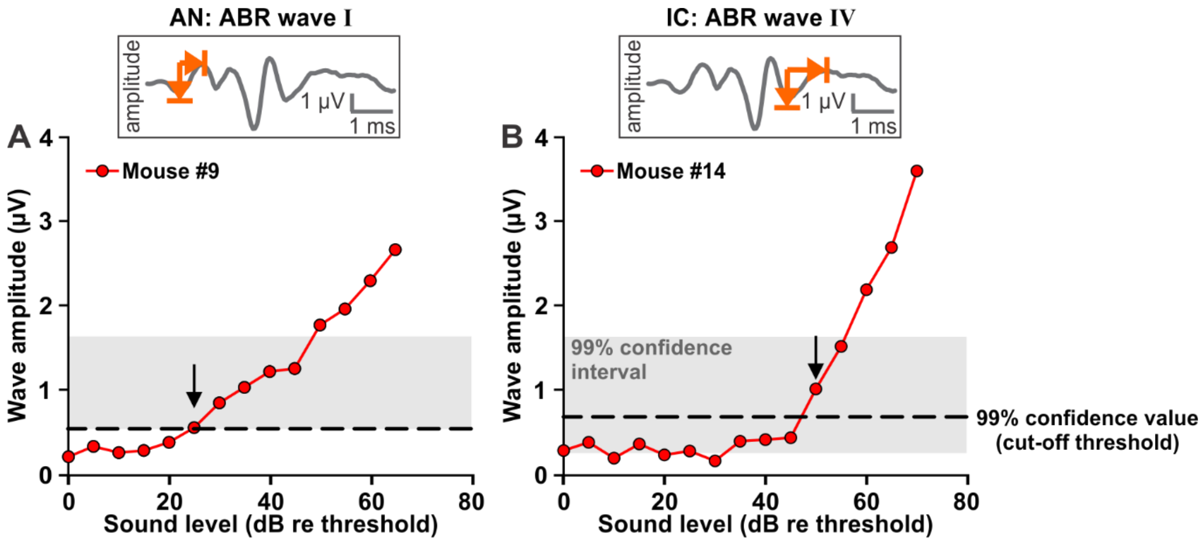

Supplementary Figure 4. Illustration of dynamic thresholding approach used for ABR amplitude growth slope analysis. (A) Example ABR wave I level-amplitude function from mouse #9. **(B)** Example ABR wave IV level-amplitude function from mouse #14. Red lines and symbols represent the individual animal's ABR wave amplitude across increasing stimulus levels (dB *re* threshold). The dashed black horizontal line indicates the 99% confidence value used as the dynamic cut-off to determine the onset of rapid amplitude growth. Gray shaded areas represent the 99% confidence interval (mean ± 99% confidence value) across the animal's amplitude values. Black arrows mark the first dB *re* threshold data point exceeding the dynamic cut-off, used as the lower bound for slope fitting.

2 Supplementary Tables

Supplementary Table 1. Number of breeding pairs used for ABR, ASSR, and AGS behavioral testing one day after hearing measurements. P20 and P32 mice were separate cohorts (no reuse of animals between ages). 'F' and 'M' indicate the number of litters female and male test animals were from, respectively, 'Total' the overall number of litters used for a given genotype × age group. 'Breeding pair' IDs indicate the litter origin of animals. P20_AGS1-4 are the same animals as in *Fmr1* KO_P20 stratified by behavioral phenotype.

	**WT**				***Fmr1* KO**			
Age	***F***	***M***	***Total***	***Breeding pair***	***F***	***M***	***Total***	***Breeding pair***
P20	3	4	4	D, E, F, G	3	3	3	A, B, C
P32	2	3	3	G, H, I	1	2	3	C, J, K
P20_AGS1	N/A	N/A	N/A	N/A	2	1	3	A, B, C
P20_AGS2	N/A	N/A	N/A	N/A	1	1	2	A, C
P20_AGS3	N/A	N/A	N/A	N/A	1	1	2	A, C
P20_AGS4	N/A	N/A	N/A	N/A	1	2	2	B, C

Supplementary Table 2. Number of breeding pairs used for brain extraction and cFos staining following AGS. This cohort was independent from animals shown in Supplementary Table 1. 'F' and 'M' indicate the number of litters female and male test animals were from, respectively, 'Total' the overall number of litters used for a given genotype × age group. 'Breeding pair' IDs indicate the litter origin of animals.

	**WT**				***Fmr1* KO**			
Age	***F***	***M***	***Total***	***Breeding pair***	***F***	***M***	***Total***	***Breeding pair***
P20	2	2	2	L, M	1	1	1	O
P32	1	1	1	N	2	1	2	P, Q

Supplementary Table 3. Statistical comparisons of behavioral phenotype from *Fmr1* KO_P20 mice of the following four AGS categories: no response (*n*=4), wild running (*n*=3), seizure (*n*=3), or respiratory arrest (*n*=5). This table is associated with data in Fig. 2.

Source of Variation	***P value***	***P value summary***	***F (DFn, DFd)***	***ηp2***
Breeding pair	ns	0.70	F(2, 9) = 0.37	0.076
Sex	ns	0.47	F(1, 9) = 0.55	0.058
Breeding pair:Sex	ns	0.28	F(2, 9) = 1.44	0.243

Two-way ART ANOVA, *p* values, ns not significant.

Supplementary Table 4. Statistical comparisons of ABR thresholds from mice of the following four groups: WT_P20 (*n*=16), *Fmr1* KO_P20 (*n*=14-15), WT_P32 (*n*=10), and *Fmr1* KO_P32 (*n*=12). The thresholds for one *Fmr1* KO_P20 animal at 4 and 32 kHz were excluded from analysis because the acoustic stimuli were presented in 10 dB instead of 5 dB steps. This table is associated with data in Fig 3A.

Parameter	***Source of Variation***	***P value***	***P value summary***	***F (DFn, DFd)***	***ηp2***
ABR threshold	Genotype	0.31	ns	F(1, 44.59) = 1.02	0.022
	Sex	0.29	ns	F(1, 44.59) = 1.10	0.024
	Age	<0.001	***	F(1, 44.54) = 22.76	0.338
	Frequency	<0.001	***	F(2, 88.49) = 398.98	0.900
	Genotype:Sex	0.60	ns	F(1, 44.58) = 0.26	0.006
	Genotype:Age	0.36	ns	F(1, 44.58) = 0.83	0.018
	Sex:Age	0.06	ns	F(1, 44.56) = 3.54	0.074
	Genotype:Frequency	0.01	*	F(2, 88.53) = 4.70	0.096
	Sex:Frequency	0.20	ns	F(2, 88.53) = 1.59	0.035
	Age:Frequency	0.01	*	F(2, 88.53) = 4.11	0.085
	Genotype:Sex:Age	0.21	ns	F(1, 44.59) = 1.58	0.034
	Genotype:Sex:Frequency	0.44	ns	F(2, 88.52) = 0.82	0.018
	Genotype:Age:Frequency	0.62	ns	F(2, 88.53) = 0.46	0.010
	Sex:Age:Frequency	0.73	ns	F(2, 88.53) = 0.31	0.007
	Genotype:Sex:Age:Frequency	0.23	ns	F(2, 88.54) = 1.49	0.033

Mixed effects ART ANOVA, *p* values, *$p < 0.05$, ***$p < 0.001$, ns not significant.

Supplementary Table 5. *Post hoc* pairwise comparisons of ABR thresholds for factor Frequency from mice of the following four groups: WT_P20 (*n*=16), *Fmr1* KO_P20 (*n*=14), WT_P32 (*n*=10), and *Fmr1 KO_P32* (*n*=12).This table is associated with data in Fig. 3A.

Parameter	*Contrast*	*Estimate*	*SE*	*DF*	*t*	*Adjusted P Value*	*P value summary*
Frequency (kHz)	11.3 - 32	-45.24	3.54	88.64703	-12.78	<0.001	***
	11.3 - 4	-99.89	3.54	88.64703	-28.21	<0.001	***
	32 - 4	-54.65	3.54	88.19087	-15.40	<0.001	***

ART-C Tukey's multiple comparisons test, *p* values, ****p* < 0.001, ns not significant.

Supplementary Table 6. *Post hoc* pairwise comparisons of ABR thresholds at 32 kHz from mice of the following five groups: WT_P20 (*n*=16), *Fmr1* KO_P20 AGS1 (*n*=4), AGS2 (*n*=3), AGS3 (*n*=2), AGS4 (*n*=5). This table is associated with data in Fig. 3B.

Contrast	**Mean rank difference**	*r*	*Z*	*Adjusted P Value*	*P value summary*
AGS1 vs AGS2	-6.13	0.37	0.93	>0.99	ns
AGS1 vs AGS3	-6.63	0.45	0.89	>0.99	ns
AGS1 vs AGS4	-5.53	0.34	0.96	>0.99	ns
AGS1 vs WT	4.41	0.21	0.92	>0.99	ns
AGS2 vs AGS3	-0.50	0.10	0.06	>0.99	ns
AGS2 vs AGS4	0.60	0.15	0.10	>0.99	ns
AGS2 vs WT	10.53	0.47	1.95	0.51	ns
AGS3 vs AGS4	1.10	0.20	0.15	>0.99	ns
AGS3 vs WT	11.03	0.42	1.71	0.86	ns
AGS4 vs WT	9.93	0.50	2.26	0.23	ns

Dunn's multiple comparisons test, *p* values, ***p* < 0.01, ns not significant.

Supplementary Table 7. Exact *n* numbers per genotype, sex, and dB *re* threshold level for the P20 cohort. The table lists the number of male (M), female (F), and total animals contributing ABR wave amplitude and latency data at each sound level for each stimulus frequency (4 kHz, 11.3 kHz, and 32 kHz). This table is associated with data in Fig. 4, 6 and Supplementary Fig. 1.

**P20**		**WT**			***Fmr1* KO**		
Frequency	***dB re threshold***	***F***	***M***	***Total***	***F***	***M***	***Total***
4 kHz	0	9	7	16	7	7	14
	5	9	7	16	7	7	14
	10	9	7	16	7	7	14
	15	9	7	16	7	7	14
	20	9	7	16	7	7	14
	25	8	7	15	7	7	14
	30	8	7	15	6	7	13
	35	7	5	12	3	7	10
11.3 kHz	0	9	7	16	7	8	15
	5	9	7	16	7	8	15
	10	9	7	16	7	8	15
	15	9	7	16	7	8	15
	20	9	7	16	7	8	15
	25	9	7	16	7	8	15
	30	9	7	16	7	8	15
	35	9	7	16	7	8	15
	40	9	7	16	7	8	15
	45	9	7	16	7	8	15
	50	9	7	16	7	8	15
	55	9	7	16	7	7	14
	60	8	6	14	7	7	14
	65	8	4	12	5	6	11
	70	5	3	8	3	3	6

32 kHz	0	9	7	16	7	7	14
	5	9	7	16	7	7	14
	10	9	7	16	7	7	14
	15	9	7	16	7	7	14
	20	9	7	16	7	7	14
	25	9	7	16	7	7	14
	30	9	7	16	7	7	14
	35	9	7	16	7	7	14
	40	8	7	15	7	7	14
	45	8	7	15	5	6	11
	50	8	6	14	3	5	8

Supplementary Table 8. Exact *n* numbers per genotype, sex, and dB *re* threshold level for the P32 cohort. The table lists the number of male (M), female (F), and total animals contributing ABR wave amplitude and latency data at each sound level for each stimulus frequency (4 kHz, 11.3 kHz, and 32 kHz). This table is associated with data in Fig. 4-6 and Supplementary Fig. 1.

**P32**		**WT**			***Fmr1* KO**		
Frequency	***dB re threshold***	***F***	***M***	***Total***	***F***	***M***	***Total***
4 kHz	0	6	4	10	6	6	12
	5	6	4	10	6	6	12
	10	6	4	10	6	6	12
	15	6	4	10	6	6	12
	20	6	4	10	6	6	12
	25	6	4	10	6	6	12
	30	6	4	10	6	6	12
	35	5	4	9	6	4	10
11.3 kHz	0	6	4	10	6	6	12
	5	6	4	10	6	6	12
	10	6	4	10	6	6	12
	15	6	4	10	6	6	12
	20	6	4	10	6	6	12
	25	6	4	10	6	6	12
	30	6	4	10	6	6	12
	35	6	4	10	6	6	12
	40	6	4	10	6	6	12
	45	6	4	10	6	6	12
	50	6	4	10	6	6	12
	55	6	4	10	6	6	12
	60	6	4	10	6	6	12
	65	6	4	10	6	5	11
32 kHz	0	6	4	10	6	6	12
	5	6	4	10	6	6	12
	10	6	4	10	6	6	12
	15	6	4	10	6	6	12
	20	6	4	10	6	6	12
	25	6	4	10	6	6	12
	30	6	4	10	6	6	12
	35	6	4	10	6	6	12
	40	6	4	10	6	6	12
	45	6	4	10	6	6	12
	50	6	4	10	6	6	12
	55	6	3	9	6	6	12
	60	6	3	9	4	4	8

Supplementary Table 9. Statistical comparisons of ABR wave latencies (leading negative peaks, AGS1, 3, 4: 0 to 70 dB re threshold; AGS2: 0 to 65 dB re threshold, at 11.3 kHz) from mice of the following four groups: *Fmr1* KO_P20 AGS1 (*n*=4), AGS2 (*n*=3), AGS3 (*n*=3), AGS4 (*n*=5). Please note that all *post hoc* multiple comparisons for

interactions involving sound level (dB *re* threshold) were not significant when matched for sound levels (not shown). This table is associated with data in Supplementary Fig. 2.

Parameter	*Source of Variation*	*P value*	*P value summary*	*F (DFn, DFd)*	*ηp2*
Wave I	AGS	0.58	ns	F(3, 17.81) = 0.66	0.101
	dB.re.Thr	<0.001	***	F(14, 140.03) = 138.48	0.933
	AGS: dB.re.Thr	0.48	ns	F(41, 140.02) = 0.99	0.226
Wave II	AGS	0.45	ns	F(3, 18.36) = 0.9	0.129
	dB.re.Thr	<0.001	***	F(14, 140.03) = 182.02	0.948
	AGS: dB.re.Thr	0.79	ns	F(41, 140.04) = 0.79	0.189
Wave III	AGS	0.70	ns	F(3, 18.11) = 0.46	0.072
	dB.re.Thr	<0.001	***	F(14, 140.03) = 95.05	0.905
	AGS: dB.re.Thr	0.07	ns	F(41, 140.03) = 1.41	0.292
Wave IV	AGS	0.27	ns	F(3, 18.71) = 1.38	0.182
	dB.re.Thr	<0.001	***	F(14, 140.09) = 81.39	0.891
	AGS: dB.re.Thr	0.02	*	F(41, 140.08) = 1.58	0.317

Mixed effects ART ANOVA, *p* values, *$p < 0.05$, ***$p < 0.001$, ns not significant.

Supplementary Table 10. P values for effects on ABR level-amplitude and level-negative peak latency functions at 4 and 32 kHz for *Fmr1* KO_P20 of the following two audiogenic seizure behavioral subgroups: no response (AGS1, *n*=4), and wild running/tonic-clonic seizure/respiratory arrest (AGS2-4, *n*=10; pooled because AGS3 at 4 and 32 kHz *n*=2). Please note that all *post hoc* multiple comparisons for interactions involving sound level (dB *re* threshold) were not significant when matched for sound levels.

Fmr1 KO_P20		*4 kHz*		*32kHz*	
Parameter	*Source of Variation*	*Amplitude*	*Latency*	*Amplitude*	*Latency*
Wave I	AGS	0.60	0.47	0.49	0.45
	dB.re.Thr	<0.001***	<0.001***	<0.001***	<0.001***
	AGS:dB.re.Thr	0.13	0.44	0.74	0.66
Wave II	AGS	0.43	0.74	0.11	0.56
	dB.re.Thr	<0.001***	<0.001***	<0.001***	>0.99
	AGS:dB.re.Thr	0.29	0.31	0.71	0.69
Wave III	AGS	0.93	0.50	0.06	0.34
	dB.re.Thr	<0.001***	<0.001***	<0.001***	<0.001***
	AGS:dB.re.Thr	0.57	0.77	0.53	0.86
Wave IV	AGS	0.31	0.36	0.93	0.96
	dB.re.Thr	<0.001***	<0.001***	<0.001***	<0.001***
	AGS:dB.re.Thr	0.71	0.03*	0.45	0.70

Mixed effects ART ANOVA, *p* values, *$p < 0.05$, ***$p < 0.001$

Supplementary Table 11. P values for effects on ABR level-amplitude and level-negative peak latency functions at 4 and 32 kHz for the following two groups: WT_P20 (*n*=16) and *Fmr1* KO_P20 (*n*=15). Please note that all *post hoc* multiple comparisons for interactions involving sound level (dB *re* threshold) were not significant when matched for sound levels. At 32 kHz, Wave III latencies were significantly slower and wave IV amplitudes lower in *Fmr1* KO_P20 compared to WT_P20 mice, possibly due to the more pronounced delay in maturation at this frequency, as identified in ABR thresholds (Fig. 3). However, there was no difference within *Fmr1* KO_P20 mice when stratified for AGS phenotype (Supplementary Table 10).

P20		*4 kHz*		*32kHz*	
Parameter	*Source of Variation*	*Amplitude*	*Latency*	*Amplitude*	*Latency*
Wave I	Genotype	0.85	0.80	0.39	0.15
	Sex	0.60	0.29	0.81	0.93
	dB.re.Thr	<0.001***	<0.001***	<0.001***	<0.001***
	Genotype:Sex	0.60	0.61	0.32	0.82
	Genotype:dB.re.Thr	0.70	0.13	0.47	<0.001***
	Sex:dB.re.Thr	0.78	0.74	0.93	0.02*
	Genotype:Sex:dB.re.Thr	0.97	>0.99	0.08	0.37
Wave II	Genotype	0.14	0.66	0.59	0.18
	Sex	0.25	0.12	0.76	0.97

	dB.re.Thr	<0.001***	<0.001***	<0.001***	<0.001***
	Genotype:Sex	0.75	0.64	0.72	0.54
	Genotype:dB.re.Thr	0.02*	0.003**	0.04*	0.63
	Sex:dB.re.Thr	0.93	0.005**	0.56	0.30
	Genotype:Sex:dB.re.Thr	0.02*	0.004**	0.32	0.72
Wave III	Genotype	0.79	0.86	0.86	0.009**
	Sex	0.57	>0.99	0.87	0.27
	dB.re.Thr	<0.001***	<0.001***	<0.001***	<0.001***
	Genotype:Sex	0.59	0.95	0.44	0.38
	Genotype:dB.re.Thr	0.95	0.05	0.005**	0.75
	Sex:dB.re.Thr	0.04*	0.08	0.90	0.96
	Genotype:Sex:dB.re.Thr	0.25	0.49	0.64	0.57
Wave IV	Genotype	0.93	0.46	0.03*	0.21
	Sex	0.97	0.46	0.97	0.33
	dB.re.Thr	<0.001***	<0.001***	<0.001***	<0.001***
	Genotype:Sex	0.72	0.13	0.13	0.65
	Genotype:dB.re.Thr	0.77	0.01*	0.11	0.52
	Sex:dB.re.Thr	0.76	0.52	>0.99	0.03*
	Genotype:Sex:dB.re.Thr	0.47	0.81	0.04*	0.76

Mixed effects ART ANOVA, *p* values, *$p < 0.05$, **$p < 0.01$, ***$p < 0.001$

Supplementary Table 12. P values for effects on ABR level-amplitude and level-negative peak latency functions at 4 and 32kHz for the following two groups: WT_P32 (*n*=10) and *Fmr1* KO_P32 (*n*=12). Please note that all *post hoc* multiple comparisons were not significant, except for ABR wave I latency at 32 kHz for interaction Genotype:Sex, Supplementary Table 13. This table is associated with Fig. 5.

P32		***4 kHz***		***32kHz***	
Parameter	***Source of Variation***	***Amplitude***	***Latency***	***Amplitude***	***Latency***
Wave I	Genotype	0.007**	0.97	0.26	0.95
	Sex	0.73	0.14	0.33	0.02*
	dB.re.Thr	<0.001***	<0.001***	<0.001***	<0.001***
	Genotype:Sex	0.07	0.59	0.78	0.02*
	Genotype:dB.re.Thr	<0.001***	0.83	0.19	0.01*
	Sex:dB.re.Thr	0.14	0.23	0.59	0.04*
	Genotype:Sex:dB.re.Thr	<0.001***	0.18	0.42	<0.001***
Wave II	Genotype	>0.99	0.37	0.91	0.71
	Sex	0.74	0.31	0.58	0.06
	dB.re.Thr	<0.001***	<0.001***	<0.001***	<0.001***
	Genotype:Sex	0.01*	0.50	0.49	0.12
	Genotype:dB.re.Thr	0.23	0.03*	0.88	0.29
	Sex:dB.re.Thr	0.86	0.86	0.44	<0.001***
	Genotype:Sex:dB.re.Thr	0.003**	0.65	0.31	0.10
Wave III	Genotype	0.29	0.93	0.13	0.74
	Sex	0.85	0.72	0.83	0.06
	dB.re.Thr	<0.001***	<0.001***	<0.001***	<0.001***
	Genotype:Sex	0.08	0.71	0.33	0.09
	Genotype:dB.re.Thr	0.02*	0.83	0.08	0.03*
	Sex:dB.re.Thr	0.02*	0.43	0.89	0.97
	Genotype:Sex:dB.re.Thr	0.63	0.64	0.13	0.96
Wave IV	Genotype	0.20	0.41	0.23	0.28
	Sex	0.19	0.81	0.44	0.08
	dB.re.Thr	<0.001***	<0.001***	<0.001***	<0.001***
	Genotype:Sex	0.30	0.76	0.83	0.35
	Genotype:dB.re.Thr	0.94	>0.99	0.68	0.20
	Sex:dB.re.Thr	0.006**	0.05	0.57	0.49
	Genotype:Sex:dB.re.Thr	0.77	0.03*	0.93	0.90

Mixed effects ART ANOVA, *p* values, *$p < 0.05$, **$p < 0.01$, ***$p < 0.001$

Supplementary Table 13. *Post hoc* pairwise comparisons of ABR wave I negative peak latency at 32 kHz for interaction Genotype:Sex for mice of the following groups: WT_P32 (*n*=10) and *Fmr1* KO_P32 (*n*=12). This table is associated with Fig. 5.

Contrast	*Estimate*	*SE*	*DF*	*t*	*Adjusted P Value*	*P value summary*
WT,F - WT,M	4.98	35.33	18.01	0.14	>0.99	ns
WT,F – *Fmr1* KO,F	70.89	31.59	17.98	2.24	0.14	ns
WT,F - *Fmr1* KO,M	-42.92	31.59	17.98	-1.36	0.53	ns
WT,M - *Fmr1* KO,F	65.91	35.35	18.05	1.86	0.27	ns
WT,M - *Fmr1* KO,M	-47.9	35.35	18.05	-1.35	0.54	ns
Fmr1 KO,F - *Fmr1* KO,M	-113.81	31.61	18.03	-3.6	0.01	*

ART-C Tukey's multiple comparisons test, *p* values, *$p < 0.05$, ns not significant.

Supplementary Table 14. Statistical comparisons of ABR wave latencies (following positive peaks, 0 to 70 dB re threshold, at 11.3 kHz) from mice of the following two groups: WT_P20 (*n*=16) and *Fmr1* KO_P20 (*n*=15). Please note that all *post hoc* multiple comparisons for interactions involving sound level (dB *re* threshold) were not significant when matched for sound levels (not shown).

Parameter	*Source of Variation*	*P value*	*P value summary*	*F (DFn, DFd)*	*ηp2*
Wave I	Genotype	0.76	ns	F(1, 27.09) = 0.09	0.003
	Sex	0.45	ns	F(1, 27.09) = 0.57	0.021
	dB.re.Thr	<0.001	***	F(14, 349.1) = 589.04	0.959
	Genotype:Sex	0.43	ns	F(1, 27.09) = 0.61	0.022
	Genotype:dB.re.Thr	0.25	ns	F(14, 349.11) = 1.22	0.047
	Sex:dB.re.Thr	0.77	ns	F(14, 349.11) = 0.7	0.027
	Genotype:Sex:dB.re.Thr	0.9	ns	F(14, 349.11) = 0.55	0.022
Wave II	Genotype	0.63	ns	F(1, 27.1) = 0.23	0.009
	Sex	0.97	ns	F(1, 27.1) = 0.001	0.0001
	dB.re.Thr	<0.001	***	F(14, 349.13) = 209	0.893
	Genotype:Sex	0.56	ns	F(1, 27.1) = 0.33	0.012
	Genotype:dB.re.Thr	0.59	ns	F(14, 349.12) = 0.86	0.034
	Sex:dB.re.Thr	0.88	ns	F(14, 349.13) = 0.56	0.022
	Genotype:Sex:dB.re.Thr	0.29	ns	F(14, 349.12) = 1.17	0.045
Wave III	Genotype	0.66	ns	F(1, 27.13) = 0.18	0.007
	Sex	0.57	ns	F(1, 27.13) = 0.31	0.012
	dB.re.Thr	<0.001	***	F(14, 349.16) = 105.55	0.809
	Genotype:Sex	0.89	ns	F(1, 27.13) = 0.01	0.001
	Genotype:dB.re.Thr	<0.001	***	F(14, 349.16) = 4.9	0.164
	Sex:dB.re.Thr	<0.001	***	F(14, 349.16) = 3.1	0.111
	Genotype:Sex:dB.re.Thr	0.07	ns	F(14, 349.16) = 1.59	0.06
Wave IV	Genotype	0.61	ns	F(1, 27.12) = 0.25	0.009
	Sex	0.73	ns	F(1, 27.12) = 0.12	0.004
	dB.re.Thr	<0.001	***	F(14, 349.14) = 54.72	0.687
	Genotype:Sex	0.81	ns	F(1, 27.12) = 0.05	0.002
	Genotype:dB.re.Thr	0.39	ns	F(14, 349.15) = 1.06	0.041
	Sex:dB.re.Thr	0.94	ns	F(14, 349.15) = 0.48	0.019
	Genotype:Sex:dB.re.Thr	0.32	ns	F(14, 349.15) = 1.13	0.043

Mixed effects ART ANOVA, *p* values, ***$p < 0.001$, ns not significant.

Supplementary Table 15. Statistical comparisons of ABR wave latencies (following positive peaks, 0 to 65 dB re threshold, at 11.3 kHz) from mice of the following two groups: WT_P32 (*n*=10) and *Fmr1* KO_P32 (*n*=12). Please note that all *post hoc* multiple comparisons for interactions involving sound level (dB *re* threshold) were not significant when matched for sound levels (not shown).

Parameter	*Source of Variation*	*P value*	*P value summary*	*F (DFn, DFd)*	*ηp2*
Wave I	Genotype	0.88	ns	F(1, 17.99) = 0.02	0.001
	Sex	0.37	ns	F(1, 17.99) = 0.81	0.043
	dB.re.Thr	<0.001	***	F(13, 233) = 325.02	0.948

	Genotype:Sex	0.09	ns	F(1, 17.99) = 3.04	0.145
	Genotype:dB.re.Thr	0.96	ns	F(13, 233) = 0.41	0.023
	Sex:dB.re.Thr	0.14	ns	F(13, 233) = 1.43	0.074
	Genotype:Sex:dB.re.Thr	0.02	*	F(13, 233) = 1.98	0.100
Wave II	Genotype	0.70	ns	F(1, 17.99) = 0.14	0.008
	Sex	0.46	ns	F(1, 17.99) = 0.56	0.030
	dB.re.Thr	<0.001	***	F(13, 233) = 139.9	0.886
	Genotype:Sex	0.26	ns	F(1, 17.99) = 1.29	0.067
	Genotype:dB.re.Thr	0.95	ns	F(13, 233) = 0.44	0.024
	Sex:dB.re.Thr	0.46	ns	F(13, 233) = 0.98	0.052
	Genotype:Sex:dB.re.Thr	0.64	ns	F(13, 233) = 0.81	0.044
Wave III	Genotype	0.61	ns	F(1, 17.99) = 0.26	0.015
	Sex	0.20	ns	F(1, 17.99) = 1.7	0.087
	dB.re.Thr	<0.001	***	F(13, 233) = 25.86	0.591
	Genotype:Sex	0.73	ns	F(1, 17.99) = 0.11	0.007
	Genotype:dB.re.Thr	0.13	ns	F(13, 233) = 1.45	0.075
	Sex:dB.re.Thr	0.63	ns	F(13, 233) = 0.82	0.044
	Genotype:Sex:dB.re.Thr	0.24	ns	F(13, 233) = 1.25	0.065
Wave IV	Genotype	0.33	ns	F(1, 17.99) = 0.99	0.052
	Sex	0.52	ns	F(1, 17.99) = 0.41	0.023
	dB.re.Thr	<0.001	***	F(13, 233.01) = 4.83	0.212
	Genotype:Sex	0.90	ns	F(1, 17.99) = 0.01	0.001
	Genotype:dB.re.Thr	0.21	ns	F(13, 233.01) = 1.29	0.067
	Sex:dB.re.Thr	0.44	ns	F(13, 233.01) = 1.01	0.053
	Genotype:Sex:dB.re.Thr	0.21	ns	F(13, 233.01) = 1.3	0.068

Mixed effects ART ANOVA, *p* values, *$p < 0.05$, ***$p < 0.001$, ns not significant.

Supplementary Table 16. Statistical comparisons of ABR wave latencies (following positive peaks, AGS1, 3, 4: 0 to 70 dB re threshold; AGS2: 0 to 65 dB re threshold in response to 11.3 kHz) from mice of the following four groups: *Fmr1* KO_P20 AGS1 (*n*=4), AGS2 (*n*=3), AGS3 (*n*=3), AGS4 (*n*=5). Please note that all *post hoc* multiple comparisons for interactions involving sound level (dB *re* threshold) were not significant when matched for sound levels (not shown).

Parameter	***Source of Variation***	***P value***	***P value summary***	***F (DFn, DFd)***	***ηp2***
Wave I	AGS	0.36	ns	F(3, 17.98) = 1.13	0.159
	dB.re.Thr	<0.001	***	F(14, 140.02) = 219.05	0.956
	AGS: dB.re.Thr	>0.99	ns	F(41, 140.02) = 0.52	0.133
Wave II	AGS	0.91	ns	F(3, 18.45) = 0.16	0.027
	dB.re.Thr	<0.001	***	F(14, 140.06) = 104.7	0.913
	AGS: dB.re.Thr	0.27	ns	F(41, 140.05) = 1.14	0.251
Wave III	AGS	0.62	ns	F(3, 18.54) = 0.6	0.089
	dB.re.Thr	<0.001	***	F(14, 140.06) = 29.84	0.749
	AGS: dB.re.Thr	0.04	*	F(41, 140.05) = 1.5	0.306
Wave IV	AGS	0.62	ns	F(3, 18.76) = 0.59	0.086
	dB.re.Thr	<0.001	***	F(14, 140.07) = 24.78	0.712
	AGS: dB.re.Thr	0.003	**	F(41, 140.07) = 1.89	0.357

Mixed effects ART ANOVA, *p* values, *$p < 0.05$, ***$p < 0.001$, ns not significant.
